# Mapping the Mutual Information Network of Enzymatic Families in the Protein Structure to Unveil Functional Features

**DOI:** 10.1371/journal.pone.0041430

**Published:** 2012-07-25

**Authors:** Daniel Aguilar, Baldo Oliva, Cristina Marino Buslje

**Affiliations:** 1 Structural Bioinformatics Group, Departament de Ciencies Experimentals i de la Salut, Universitat Pompeu Fabra, Barcelona Biomedical Research Park, Barcelona, Spain; 2 Bioinformatics Unit. Fundación Instituto Leloir, Buenos Aires, Argentina; Aberystwyth University, United Kingdom

## Abstract

Amino acids committed to a particular function correlate tightly along evolution and tend to form clusters in the 3D structure of the protein. Consequently, a protein can be seen as a network of co-evolving clusters of residues. The goal of this work is two-fold: first, we have combined mutual information and structural data to describe the amino acid networks within a protein and their interactions. Second, we have investigated how this information can be used to improve methods of prediction of functional residues by reducing the search space. As a main result, we found that clusters of co-evolving residues related to the catalytic site of an enzyme have distinguishable topological properties in the network. We also observed that these clusters usually evolve independently, which could be related to a fail-safe mechanism. Finally, we discovered a significant enrichment of functional residues (e.g. metal binding, susceptibility to detrimental mutations) in the clusters, which could be the foundation of new prediction tools.

## Introduction

Some protein functions are maintained by concerted changes of a group of residues forced to co-evolve (i.e. when a residue is mutated, other residues must change to preserve or restore the structure or function of the protein). Such is the case of enzymes, where the environment of the active site must conserve certain characteristics so that the protein maintains its function during the course of evolution [1]. It is also known that protein folds have evolved under constraints imposed by function, so their structure is robust against random mutational events, yet extremely sensitive to perturbations at key positions [2].

Likewise, functionally important residues undergo sequence variations as they evolve and form spatial clusters in the protein structure. Such clusters may be part of binding sites, catalytic sites or allosteric pathways [3]. Previous works have suggested a link between functionally important sites (for specificity or allosteric regulation) and neighbouring co-evolving residues [1], [4], [5]–[9]. Halabi *et al.*, using sequence-based analysis, introduced the concept of groups of correlated amino acids that evolved quasi-independently, called *sectors*. Strikingly, those sectors were observed to be physically in contact in the 3D structure [10].

In addition, the information within a protein must be transmitted, at least partly, between residues in physical contact, some being important to maintain a short path in the distance network [2], [11]. Thus, it is reasonable to consider a protein as an undirected network of contacting residues. Decomposing protein structures into modules of densely-interconnected residues using this kind of network representations has been useful to explain allosteric communication [12].

In this work we analyse the mutual information networks between residues (MIN) in 187 families of enzymes and describe the relationship between co-evolution and the 3D structure of the protein. We introduce a new concept, the analysis of MI3D clusters which combine both evolutionary and three-dimensional information. In accordance with Halabi *et al.*, we observed that networks of co-evolving residues tend to be close, forming a *sector* (which we called *MI3D cluster*) when mapped onto the 3D structure [10]. Furthermore, we found that, amongst the many MI3D clusters usually present in a protein domain, those containing catalytic residues have distinguishable network properties. This finding could be used to predict such catalytic residues. Finally, we measured the enrichment of the clusters in residues with different functionalities, e. g: catalytic activity, metal binding, and susceptibility to detrimental mutations.

## Results and Discussion

### Are Mutual Information Networks and Distance Networks Topologically Different?

We created the Mutual Information Networks (MINs) by connecting residues with a mutual information value > = 6 since such value was determined to be indicative of a significant evolutionary relationship [13] (see Methods). The Distance Networks (DNs) were created by connecting any two residues if any heavy atom of each was closer than 5 Å. As an example, figure S1 shows the MIN and DN for the Pfam family PF00884.

**Figure 1:**
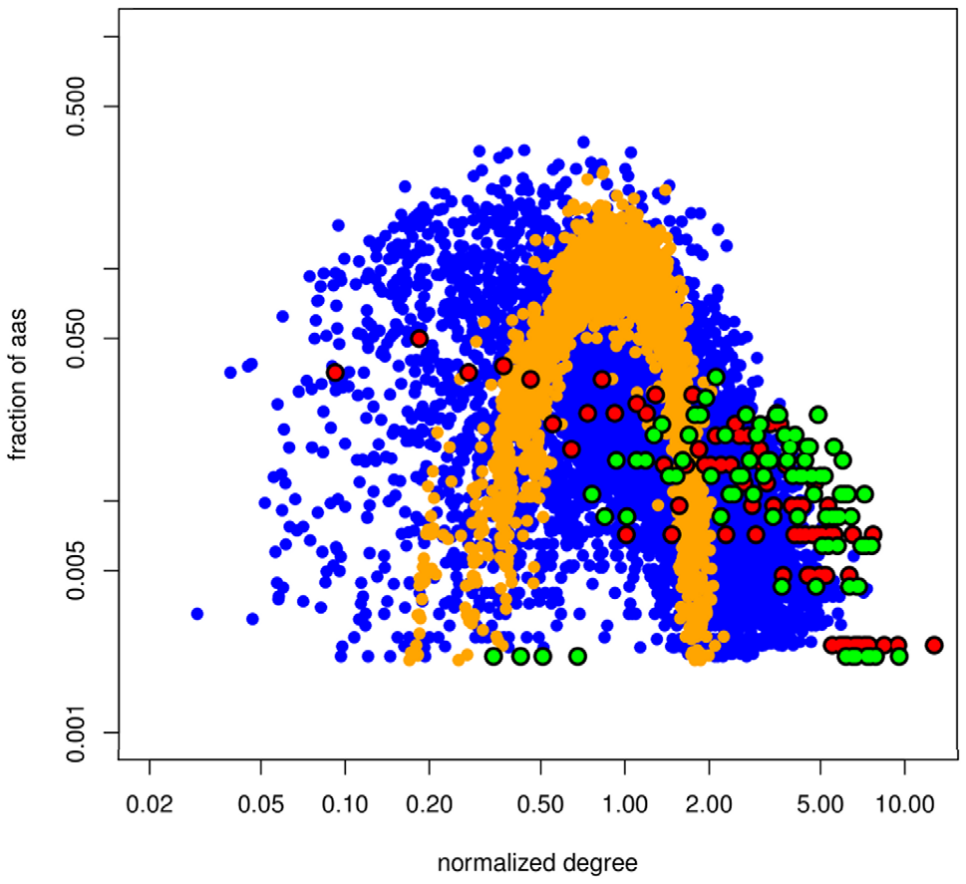
Degree distribution of the MIN and DN. Fraction of amino acids versus the normalized degree in the MINs (blue dots) and DNs (orange dots). Degrees were normalized with respect to the average degree. All DNs followed a Poisson distribution (α = 0.01, KS test). 43.8% of MINs followed a power-law distribution and 29.9% followed a Poisson distribution (α = 0.01). Red dots: normalized degree distribution in the MIN for Pfam family PF01432, showing a truncated power-law distribution. Green dots: normalized degree distribution in the MIN for Pfam family PF00118. Logarithmic scale.

We observed that the topological properties of MINs and DNs cannot be inferred from one another. The degree distribution of the DN of any protein structure follows a bell-like Poisson distribution as would be expected for a statistically homogeneous random model (Figure 1). This has been observed by other authors, and has been attributed to a restriction in the number of residues occupying a volume in the protein space [14]–[16]. The distribution degree of the MINs displays a mixture of distributions ranging from fast-decaying power-law to Poissonian (Figure 1). Since co-evolution demands some degree of physical proximity [17], [18], deviations from a purely scale-free architecture may be due to a limit in the possible number of neighbours that a node can have. Also, there are a number of biological factors influencing the ability of two residues to co-evolve (e.g. their functional roles, their biochemical nature, their structural surroundings, etc.), which can possibly hinder the existence of a large number of simultaneously co-evolving residues. This would prevent the existence of nodes with distinctly large numbers of neighbours in the MINs, thus truncating the characteristic long tails in power-law degree distributions.

**Figure 2:**
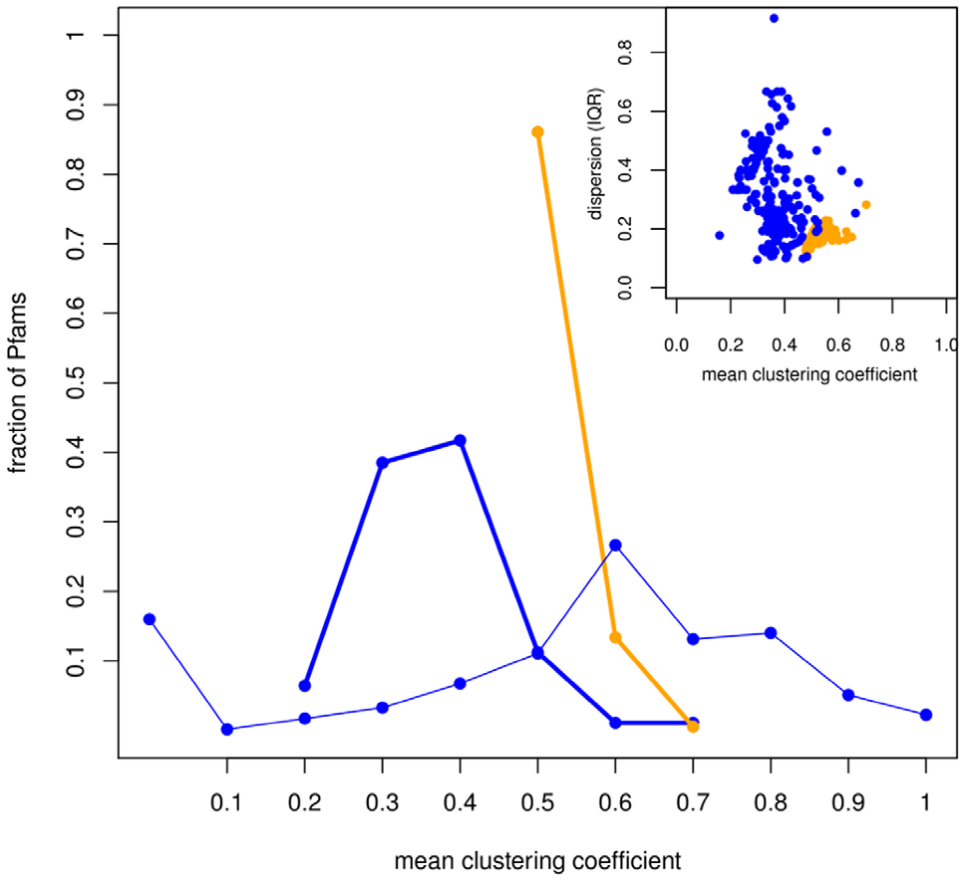
Distribution of the mean clustering coefficient for MINs, DNs, MI clusters. Thick blue line: MINs. Thick orange line: DNs. Thin blue line: MI clusters. The average clustering coefficient is 0.365 for MINs and 0.514 for DNs. Both values are statistically larger than random expectation with *p*-value <2.2*10^−16^. Inset: dispersion of the data measured as Inter-Quantile Range (IQR). Only the giant components of the MINs and DNs are considered.

The clustering coefficient distribution of the MINs and DNs is shown in Figure 2. The mean values for the distributions are 0.365 for MINs, and 0.514 for DNs. This implies networks with very dense regions, which is in line with the observations of some other biological networks [19]. However, the dispersion of the clustering coefficient in MINs is significantly larger than in DNs (*p*-value <2.2*10^−16^, KS test; Figure 2, inset). This large dispersion is characteristic of modular architectures [20]. This means that residues in the MINs tend to form small clusters where all residues evolutionarily influence one another, while there is very little evolutionary dependence between clusters. Finally, analysis of clustering coefficient and characteristic path length shows that MINs and DNs show a small-world structure as described by Watts and Strogatz in their seminal work [21]: characteristic path length *L* > =  *L_random_* and clustering coefficient *C* >> *C_random_*, where *C_random_* and *L_random_* are parameters for random networks (details in text S1). We also compared MINs and DNs to regular networks (see text S1). Our results for DNs agree with the observations of Vendruscolo *et al.* for protein structures [22]. Results for MINs are similar to those observed by Chakrabarti and Panchenko [23]. The only difference is that, in our case, *C* < *C_regular_* in most MINs (95.6%), while in theirs *C* > *C_regular_* on average. Two possible explanations for this discrepancy are: (1) Chakrabarti and Panchenko used a lower number of aligned sequences in order to calculate MI values (which can be source of bias [24]), and (2) a different method for mutual information threshold calculation was used, which in our case produced sparser networks.

### Are Groups of Co-evolving Amino Acids Close in the 3D Structure?

The MCL algorithm was used to identify clusters of co-evolving residues in the MINs (see Methods), hereinafter called *MI clusters*. These MI clusters contain groups of co-evolving residues, regardless their distance in the 3D structure of the protein. Figure S2 shows the modularity of the MINs (see Methods).

It has been demonstrated that co-evolving residues are distributed in a particular fashion in the 3D structure [1]. For instance, two distant residues can actually belong to the same MI cluster (this is, they co-evolve) while two neighbouring residues may evolve separately despite their physical proximity. We investigated the spatial arrangement of MI clusters by mapping them onto the DN (i.e. onto the 3D structure of the reference protein, see Methods). The resulting clusters were called *MI3D clusters* (see Methods). These MI3D clusters contain groups of co-evolving residues which are close (<5 Å) in the 3D structure of the protein.

About 80% of the MI clusters generate MI3D clusters once mapped onto the DN (clusters with less than four residues were not considered in our analysis). The number of MI3D clusters per Pfam and their size distribution are shown in figures S3 and S4. About 75% of MI clusters are preserved as a single cluster and only about 6% of the MI clusters are split into two or more isolated MI3D clusters when mapped onto the structure of the protein. These results confirm that groups of co-evolving amino acids tend to be spatially close [25], [26] Although previous studies have shown that co-evolution does also occur between non-contacting residues [27], [28], our findings show that there is a link between co-evolution and physical contact. However, the fact that about 6% of MI clusters are broken into several isolated MI3D clusters means that a pair of residues can actually co-evolve even though they are neither in physical contact nor “connected” by single-linkage of co-evolving residues. A common selective pressure in two separated areas of the protein could explain this observation (e.g. two interaction patches, allosterism, etc.).

### Catalytic MI3D Clusters

We next identified those MI3D clusters which either contain a catalytic residue or are close to one (<5 Å). We called those clusters *catalytic MI3D clusters*. Figures S5 and S6 show that, on average, non-catalytic MI3D clusters are more than twice as frequent as catalytic MI3D clusters. This can be explained given the singularity of the catalytic site. Other noticeably feature is that, within a Pfam domain, the catalytic MI3D clusters are, on average, 4-fold larger than the non-catalytic ones (see figure S7).

**Figure 3:**
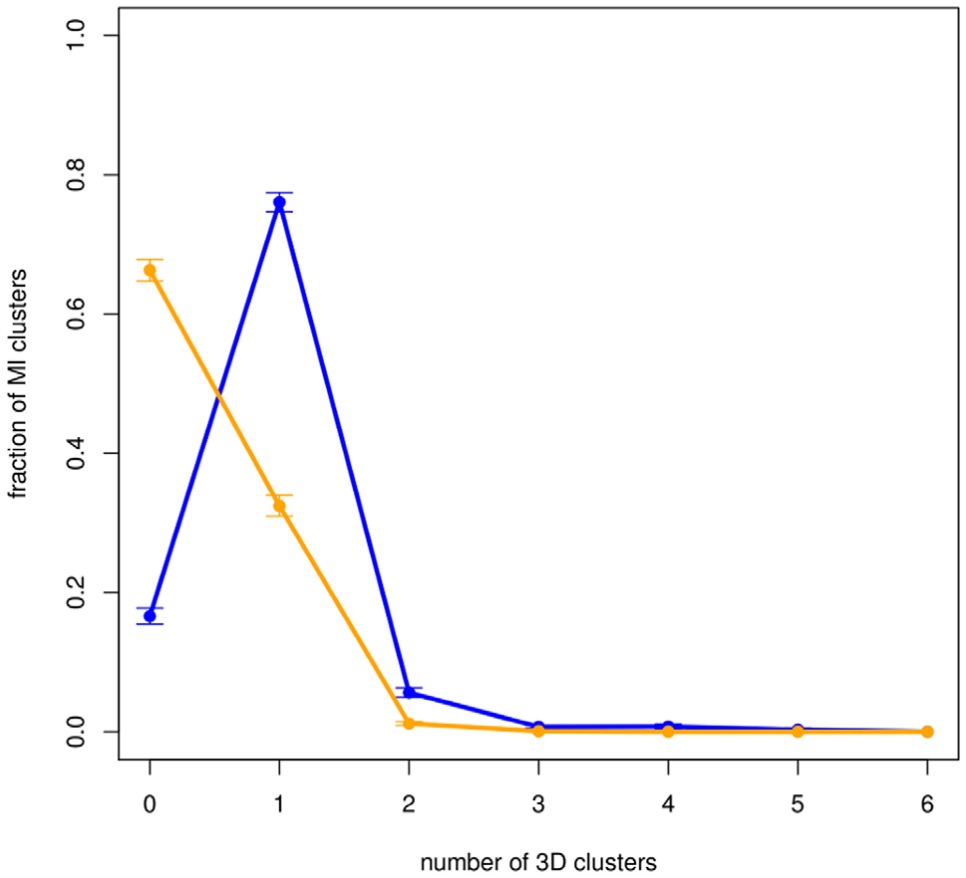
Distribution of the number of 3D clusters generated by mapping the MI cluster onto the 3D structure. Blue line: all MI3D clusters. Orange line: only catalytic MI3D clusters. 74.1% of MI clusters are preserved as a single MI3D cluster after mapping (93.1% according to random expectation). 25.9% of MI clusters produce one catalytic MI3D cluster after mapping (92.0% according to random expectation).

Although the majority of MI clusters (72.9%) do not produce catalytic MI3D clusters (but produce non-catalytic MI3D clusters), it is worth noting that, when they do, they mostly produce only one (Figure 3). As expected, considering that they originate from different MI clusters, catalytic MI3D clusters within the same Pfam are less likely to co-evolve than random expectation (figure S8).

Taking into account that there are, on average, more than 3 catalytic MI3D clusters per Pfam, this implies that catalytic MI3D clusters usually evolve independently despite their physical proximity. This is suggestive of either an integrative process (which “connects” different parts of the protein to the catalytic site) or a fail-safe mechanism where mutual information connects functional residues, for instance, in the event of losing a catalytic MI3D cluster (e.g. due to a mutation).

Furthermore, we observed that a catalytic and a non-catalytic MI3D cluster can actually co-evolve, as 23% of the Pfams with catalytic and non-catalytic MI3D clusters, at least one catalytic and one non-catalytic MI3D cluster are derived from the same MI cluster (figure S9).This suggests the presence of catalytic subsites, e.g. positions that determine specificity or other necessary residues for the accomplishment of the catalysis (e. g. allosteric sites).

### Prediction of Functional Sites Using MI Information

It is reasonable to expect important residues to be subject to co-evolutionary pressures. In agreement with the idea of using communities of correlated amino acids to uncover sets of residues defining functional characteristics in a protein family [29], we investigated whether MI clusters were enriched in residues relevant for the enzymatic activity, such as metal-binding residues and residues forming the active site. Our results indicate that the UniProt features ACT_SITE, BINDING, MUTAGEN and METAL are significantly over-represented in MI clusters (Table 1). We also investigated if each residue’s degree in MI clusters (i.e. the number of residues to which it is linked by co-evolution) has any influence on its functional relevance. We grouped the residues of each Pfam’s MI clusters into three ranges according to their degree (see Methods). As shown in Table 2, ACT_SITE, BINDING and METAL features are significantly more frequent in residues with large degrees than in those with medium and low degree. Similarly, we calculated that METAL and ACT_SITE features are significantly more frequent in residues with a large clustering coefficient.

**Table 1:** Enrichment of functional features in MI clusters and MI3D clusters.

	MI clusters	MI3D clusters	catalytic MI3D clusters	non-catalytic MI3D clusters
	Odds ratio	Odds ratio	Odds ratio	Odds ratio
**ACT_SITE**	1.345*	1.341*	2.457*	0.141*
**BINDING**	1.312*	1.428*	2.431*	0.446
**METAL**	1.333*	2.210*	3.292*	0.166
**SITE**	1.237	1.264	3.011	0.403
**MUTAGEN**	1.652*	1.931*	2.754*	–
**MOD_RES**	1.200	0.982	1.527	0.032

Enrichment (average odds-ratio) of functional features in MI clusters, MI3D clusters, catalytic MI3D clusters and non-catalytic MI3D clusters. Stars indicate statistical significance at α = 0.01 (Fisher’s exact test).

**Table 2:** Enrichment of functional features in MI clusters associated to degree and clustering coefficient.

	Small *k*	Medium *k*	Large *k*	Signif.	Small *C*	Medium *C*	Large *C*	Signif.
**MOD_RES**	0.521	0.700	1.383	–	0.629	0.911	1.085	–
**MUTAGEN**	0.136	1.397	1.582	–	1.201	0.189	1.116	–
**METAL**	0.600	0.904	1.605	*	0.574	0.920	1.236	*
**ACT_SITE**	0.330	0.735	1.574	*	0.762	0.884	1.055	*
**BINDING**	0.311	0.513	1.843	*	0.666	1.303	0.949	–
**SITE**	0.433	1.196	1.424	–	0.664	0.929	0.980	–

Enrichment (average odds-ratio) of functional features in MI clusters associated to degree (*k*) and clustering coefficient (*C*). Stars indicate statistical significance at α = 0.01 (Kruskal-Wallis test).

### Prediction of Functional Sites Using MI and 3D Information

We next investigated whether MI3D clusters are also enriched in residues important for the enzymatic activity. As shown in Table 1, MI3D clusters were significantly enriched in residues with UniProt features ACT_SITE, BINDING, METAL and MUTAGEN as compared to MI clusters. For instance, the enrichment in metal-binding residues increases more than 2-fold as compared to random expectation. This shows the advantage of combining mutual information with structural information.

We also found that features such as ACT_SITE and BINDING are associated with residues with larger degrees (Table 3). Also, the ACT_SITE feature was slightly (but significantly) more frequent in residues of MI3D clusters with a low clustering coefficient. It is probable that they act by functionally influencing many other residues, which are not close to one another. Consequently, an alteration of these low-clustering-coefficient residues might greatly disrupt their surroundings.

**Table 3:** Enrichment of functional features in MI3D clusters associated to degree and clustering coefficient.

	Small *k*	Medium *k*	Large *k*	Signif.	Small *C*	Medium *C*	Large *C*	Signif.
**MOD_RES**	0.665	1.172	1.617	–	0.940	1.101	0.708	–
**MUTAGEN**	0.248	1.317	0.307	–	0.371	0.932	1.182	–
**METAL**	0.956	0.949	1.054	–	1.498	1.264	0.796	–
**ACT_SITE**	0.441	0.948	1.202	*	1.349	1.091	0.508	*
**BINDING**	0.714	0.930	1.381	*	1.304	1.198	0.651	–
**SITE**	0.112	0.723	1.428	–	1.349	0.969	0.294	–

Enrichment (average odds-ratio) of functional features in MI3D clusters associated to degree (*k*) and clustering coefficient (*C*). Stars indicate statistical significance at α = 0.01 (Kruskal-Wallis test).

Next, we focused on the functional enrichment in catalytic MI3D clusters, finding that UniProt features ACT_SITE, BINDING, METAL and MUTAGEN are significantly enhanced in such clusters (Table 1). This result is expected for the ACT_SITE feature, but it might be potentially predictive for the other features. ACT_SITE and BINDING are also associated to lower clustering coefficients (Table 4). The ACT_SITE feature is significantly under-represented in non-catalytic MI3D clusters (ten times less likely to occur than random chance on average). No statistical association was found between functional residues and topological parameters for non-catalytic MI3D clusters (Table 5).

**Table 4:** Enrichment of functional features in catalytic MI3D clusters associated to degree and clustering coefficient.

	Small *k*	Medium *k*	Large *k*	Signif.	Small *C*	Medium *C*	Large *C*	Signif.
**MOD_RES**	0.580	1.223	1.322	–	0.894	1.218	0.828	–
**MUTAGEN**	0.677	1.437	0.229	–	0.461	0.767	1.368	–
**METAL**	0.997	0.961	1.021	–	1.205	1.164	1.023	–
**ACT_SITE**	0.721	0.856	1.137	–	1.229	0.862	0.620	*
**BINDING**	0.930	1.024	1.253	–	1.343	1.016	0.636	*
**SITE**	0.487	1.249	0.773	–	1.137	0.980	0.405	–

Enrichment (average odds-ratio) of functional features in MI3D clusters associated to degree (*k*) and clustering coefficient (*C*). Stars indicate statistical significance at α = 0.01 (Kruskal-Wallis test).

**Table 5:** Enrichment of functional features in non-catalytic MI3D clusters associated to degree and clustering coefficient.

	Small *k*	Medium *k*	Large *k*	Signif.	Small *C*	Medium *C*	Large *C*	Signif.
**MOD_RES**	1.308	2.492	0.744	–	1.417	0.000	1.288	–
**MUTAGEN**	–	–	–	–	–	–	–	–
**METAL**	1.972	0.279	0.190	–	1.545	0.413	0.928	–
**ACT_SITE**	0.000	2.084	1.500	–	0.667	4.212	0.000	–
**BINDING**	1.032	1.047	0.523	–	0.659	1.130	1.156	–
**SITE**	0.000	0.000	2.116		1.961	0.801	0.000	–

Enrichment (average odds-ratio) of functional features in MI3D clusters associated to degree (*k*) and clustering coefficient (*C*). Stars indicate statistical significance at α = 0.01 (Kruskal-Wallis test).

### Prediction of Catalytic MI3D Clusters

**Figure 4:**
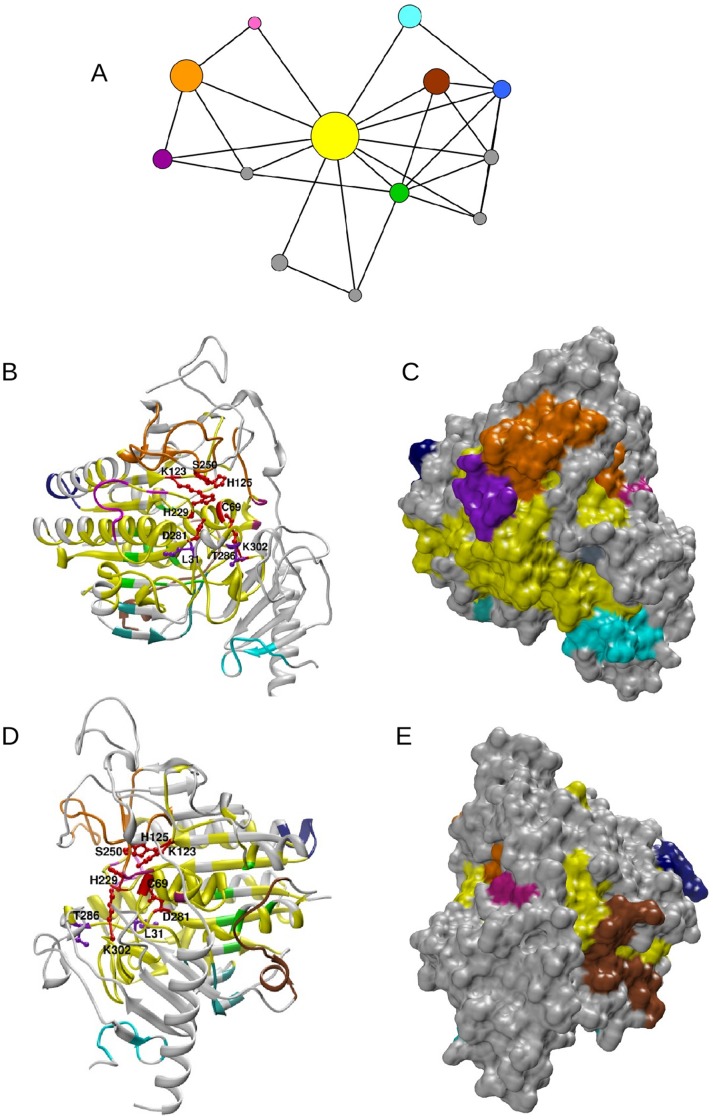
Network of MI3D clusters and mapping onto the protein structure. (A) 3DCN of the arilsulfatase A (pdb code: 1AUK chain A, PF00884). Catalytic MI3D clusters: yellow, orange and green. Non-catalytic MI3D clusters: magenta, brown, blue, cyan and pink. The size of a node in the 3DCN is proportional to the number of residues in the MI3D cluster. Clusters with less than 10 residues were coloured grey. (B) Ribbon representation of the MI3D clusters of a representative structure of PF00884. Colours are as in panel A. Catalytic residues are highlighted as red balls and sticks in the ribbon plot. Disease-related mutations are represented as violet balls and sticks. (C) Surface representation of the view in panel B. (D) Same as panel B rotated 180 degrees. (E) Surface representation of the view in panel D.

For each Pfam, a network of MI3D clusters was defined as follows: each MI3D cluster is a node, and edges between pairs of nodes exist if at least two residues (each from a different node) are closer than 5 Å. This network was named *MI3D cluster Network* (3DCN; see Methods). A graphical representation of the 3DCN and structural mapping for Pfam domain PF00884 is shown in Figure 4. Other examples are shown in figures S10 (PF01979) and S11 (PF00118).

**Figure 5:**
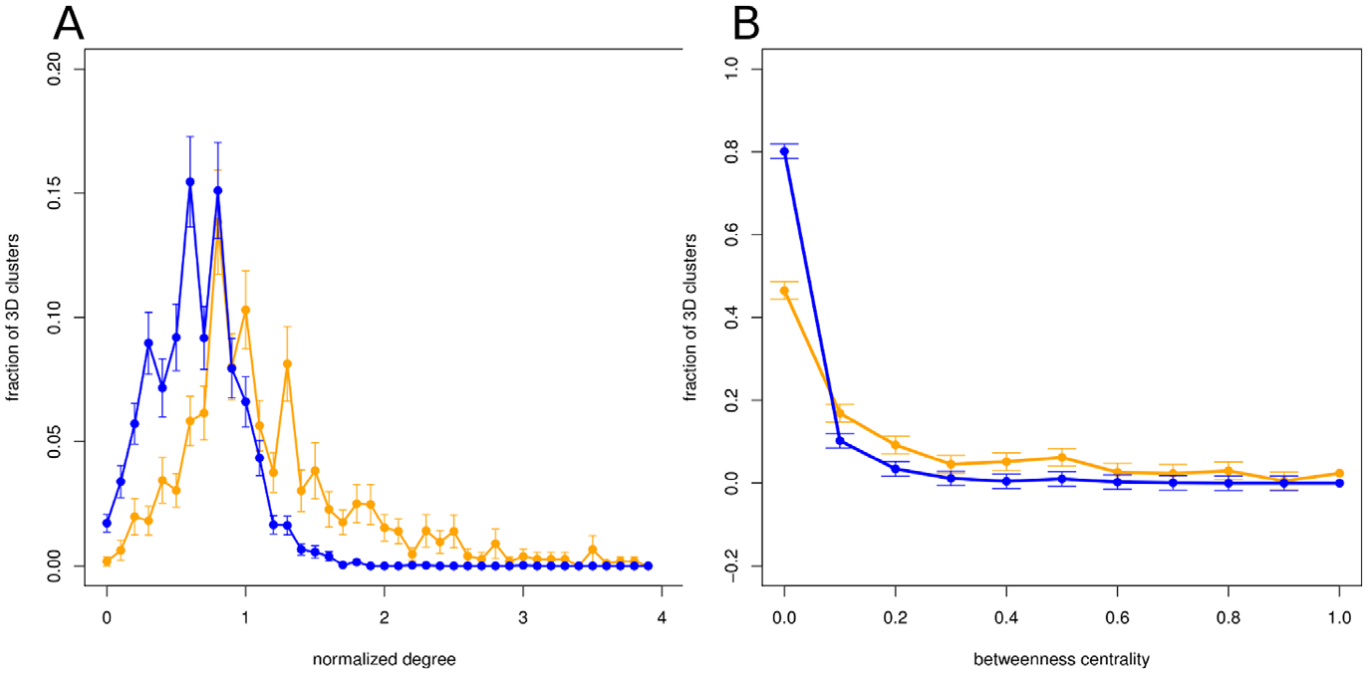
Topological parameters in the MI3D cluster network. (A) Frequency of MI3D clusters vs the normalized degree in the 3DCN. We define the normalized degree of a node as the degree divided by the average degree in the 3DCN. Orange line: catalytic MI3D clusters. Blue line: non-catalytic MI3D clusters. Mean for catalytic MI3D clusters = 1.315 (median = 1.2). Mean for non-catalytic MI3D clusters = 0.847 (median = 0.8). Both distributions are significantly different (KS test, *p*-value<2.2*10^−16^). (B) Frequency of MI3D clusters vs betweenness centrality in the 3DCN. Orange line: catalytic MI3D clusters. Blue line: non-catalytic MI3D clusters. Mean betweenness centrality for catalytic MI3D clusters = 0.159 (median = 0.059). Mean betweenness centrality for non-catalytic MI3D clusters = 0.034 (median = 0.0064). Both distributions are significantly different (KS test, *p*-value<2.2*10^−16^).

We then sought distinctive topological characteristics in the 3DCNs which could be used to identify catalytic clusters in the protein. Figure 5a shows the distribution of the normalized degrees of catalytic and non-catalytic MI3D clusters in the 3DCN. Non-catalytic MI3D clusters have significantly smaller degrees than catalytic ones, without a noticeable correlation between the degree and the size of the 3DCN (r = 0.187). Furthermore, non-catalytic MI3D clusters have, on average, significantly lower values of betweenness centrality in the 3DCN than catalytic MI3D clusters (Figure 5b). Those values are largely independent from the size of the 3DCN (r = 0.159). Also, the ratio of the betweenness centrality values of a Pfam’s catalytic and non-catalytic MI3D clusters is around 12 on average (figure S12). This suggests a central role of catalytic MI3D clusters in the distribution of information from the catalytic site to the rest of MI3D clusters.

The size, betweenness centrality and degree of MI3D clusters in the 3DCN could be used to predict catalytic MI3D clusters. We measured the performance of our method by means of an F-measure (see Methods). With optimal thresholds for the three predictors (relative size = 0.67, betweenness = 0.06, relative degree = 0.85), we achieved a 73.2 F-measure. A comparison of our results with the results if we used only spatial clusters (i.e. clusters of residues close in space) highlights the benefits of using co-evolutionary information (text S2).

### Mining OMIM Mutations in 3DCNs

We mined OMIM database searching for enzymes with specific functional words, such as *increasing* and *decreasing*, related with *action* or *activity*. For all Pfams under study, we identified 11 mutations falling within a MI3D cluster, 10 located in catalytic clusters and one in a non-catalytic MI3D cluster (table S1). We chose to analyse the mutations of the Pfam families PF00884 and PF02779, since their reference sequence is human and their 3D structure is known. The reference sequence of PF00884 is ARSA_HUMAN (UniProt accession: P15289). This Pfam family contains a N-acetylgalactosamine-4-sulfatase (Arylsulfatase B; ARSB_HUMAN; UniProt accession: P15848) and a N-acetylgalactosamine-6-sulfatase (GALNS_HUMAN; UniProt accession: P34059). The catalytic residues of ARSA_HUMAN are 69C, 123 K, 125 H, 150 S, 229 H, 281 D and 302 K. The mutation LEU72GLN in ARSB_HUMAN (OMIM code 611542) is known to cause type VI mucopolysaccharidosis, as is the mutation THR312SER in GALNS_HUMAN (OMIM code 612222). These mutations are located at 31 L and 286 T in the reference protein, respectively, both in the same catalytic MI3D cluster. Figure 4 shows the positions of both mutations, relatively close to two catalytic residues (31 L is close to 281 D and 286 T is close to 302 K).

We also found the mutation ARG183PRO of human piruvate dehydrogenase (ODPB_HUMAN; UniProt accession: P11177; OMIM code 248611) in the Pfam family PF02779. Arginine 183 corresponds to 115 R in the Pfam reference protein with known structure (PDB code 1NI4). This position was found in a catalytic MI3D cluster, therefore co-evolving with other residues close to the catalytic residues. According to OMIM, this mutation induces a structural change in the protein and impairs proper folding. According to our result, even if the protein could fold, the mutation would impair the catalytic cluster, thus impairing the activity of the enzyme. In both cases, its result would be the loss of or a deficiency in dehydrogenation of piruvate.

### Concluding Remarks

In conclusion, we have presented a study on the topology of the mutual information network and how it can be exploited to predict the location of different functional residues of enzymatic protein families. We observed that combination co-evolutionary with spatial information unveils a dense network of physically close co-evolving residues within the protein structure, which can be identified by clustering methods. The topological properties of these clustered structures suggest a role in increasing the tolerance to functional disruption and to enhance the adaptativeness of the protein. Also, these properties can be used to functionally characterize the clusters. In addition, we measured the enrichment of the clusters in functional residues, a feature that can be exploited as a prediction tool by reducing the search space when looking for functional sites. We applied the analysis in two relevant biological examples.

## Materials and Methods

### Dataset

The dataset was constructed based on the CSA database (version 2.2.11, released August 2009) [30]. CSA provides catalytic site annotation for enzymes in the PDB. Catalytic residues were defined as those residues thought to be directly involved in some aspect of the reaction catalysed by an enzyme (for a detailed description of the classification see [31]. CSA contains 968 original literature entries, which belong to 455 PFAM families [32]. We selected those families containing more than 400 unique sequences/clusters (sequences with less than 62% identity). This condition was necessary to provide a reliable estimation of MI as shown by Buslje *et al*. 2009 [13], so that we ended up with a dataset of 172 protein families, each one containing at least one PDB entry. When more than one PDB entry with catalytic site annotation was available for a given family, one reference PDB entry was selected according to the following criteria: highest sequence coverage of the Pfam MSA, the year of structure determination (preferably later than 2000) and resolution. In all cases, MSAs were gap trimmed to remove positions with gaps in the reference sequence. In addition, all positions with *>*50% gaps, as well as sequences covering *<*50% of the reference sequence length were removed, as described in [13].

### MI Calculation

Mutual Information (MI) was calculated between pairs of columns in the MSA as described in [13]. Briefly, the frequency for each amino acid pair was calculated using sequence weighting techniques and low count corrections and was compared to the expected frequency assuming that mutations between amino acids were uncorrelated. Next, the MI was calculated as a weighted sum of the log-ratios between the observed and expected amino acid pair frequencies. The APC method of Dunn *et al*. 2008 [17] was applied to reduce the background mutual information signal for each pair of residues and the MI scores were finally translated into MI *z*-scores by comparing the MI values for each pair of position with a distribution of prediction scores obtained from a large set of randomized MSAs. The *z*-score is then calculated as the number of standard deviations that the observed MI value falls above the mean value obtained from the randomized MSAs. Although the concept of *z*-score normalization was introduced by [17], [33]–[35], we use our particular score that gave better performance in both biological and *in silico-generated* benchmarks by applying APC, sequence weighting, low-count corrections and sequence permutations to calculate a sequence based *z*-score for MI distribution [13]. This combination gave the best performance, significantly improved the prediction accuracy and allows for direct comparison of information values across protein families. We found that an average sequence-based *z*-score threshold of 6.5±2.5 for a Pfam-tested benchmark (and of 6.1±1.1 for another set of biologically meaningful proteins) defined a sensitivity of 0.4 and a specificity of 0.95 [13]. Based on these results, in this work we chose a MI score threshold of 6.0.

### MI and DN Network Creation

MI networks were defined for each Pfam family as graphs G(N,E), where nodes (set N) were defined as positions in the MSA of a family (i.e. the columns of the MSA) and edges (set E) were defined between any pair of nodes with MI>6 (figure S13a). These networks were called *MI Networks* (MINs). MINs might be made of several components (disconnected subnetworks) of varying size. We focused our topological analysis on the largest component (also called giant component).

Distance networks (DN) were defined for the reference protein of every Pfam family of the dataset as graphs G′(N′,E′), where nodes (set N′) are the residues (i.e. residue numbering) of the reference protein, and edges (set E′) were defined between pairs of residues at a distance shorter than 5 Å (figure S13a). The distance between pairs of residues was defined as the smallest distance between any two heavy atoms of each residue. The value of 5 Å approximates the upper limit for attractive London-van der Waals forces [16]. Although interactions of charged atoms can occur at distances of about 8 Å (being the electrostatic energy linearly proportional to the inverse of the distance) they are not considered here. All DNs but one were composed of a single component, the exception being the result of loss of structural data in the PDB database.

### Network Clustering

Several graph-clustering methods have been devised to partition a network into clusters based on connectivity parameters. We used the MCL algorithm [36], which has been extensively used for biological network clustering and has been proved to outperform other methods [37]. Briefly, the MCL algorithm simulates random flows through a network by calculating successive powers of the associated adjacency matrix. Upon each iteration, high-flow and low-flow regions are enhanced until the process converges, partitioning the network into a set of high-flow regions surrounded by regions without any flow. The value of the inflation parameter strongly influences the number of resulting clusters. In order to calculate the optimal inflation for our networks, we used the *cml info* utility provided by the MCL software, which computes performance measures for different clusterings. For each one of our 187 MINs, we calculated the inflation value which yielded a better partition and we used it to cluster the MIN (figure S13b). The resulting clusters were termed *MI clusters*. All MI clusters with less than 4 residues were discarded.

The quality of the division was tested by calculating the modularity of the partition [38], a global parameter which can be used to test such a clustering process. Ranging between −1 and +1, positive modularity values mean that the partition of the network yielded denser subnetworks than random expectation, while negative values mean that the resulting subnetworks are not as dense as random expectation. Modularity was calculated with the *community.modularity* function of the *networkx* package in Python programming language [39].

### Network Mapping

Each MI cluster was mapped onto the DN of the reference protein. An edge between two residues was defined if they were also connected in the DN (less than 5 Å apart). The resulting subnetworks within the DNs are hereinafter defined as *MI3D clusters*. This sometimes brakes up the MI clusters into separate subnetworks (figure S13c). The MI3D clusters containing catalytic residues (or residues in close contact with any of the catalytic residues) were labelled as *catalytic MI3D clusters*. The rest of the MI3D clusters were named *non-catalytic MI3D clusters*.

### Network of MI3D Clusters

A network of MI3D clusters of a reference protein (and the corresponding Pfam) was defined as a graph G(N”,E”), where nodes were the MI3D clusters (set N”) and edges (set E”) were defined between MI3D clusters if at least one pair of residues of each cluster was in contact (distance <5 Å; figure S13d). These networks were named *MI3D cluster Networks* (3DCN).

### Topological Parameters

In order to characterize the topology of the different kinds of networks that we generated, we calculated a number of parameters (defined in table S2). Local parameters: degree, clustering coefficient. Global parameters: modularity and characteristic path length. Betweenness centrality was also used for the characterization of the *MI3D cluster networks*. Power-law and Poisson degree distributions were fitted to the MINs and DNs by means of the *fitdistr* function in R using a maximum likelihood estimation [40], and then compared by means of a Kolmogorov-Smirnov test. All topological parameters were calculated using the *networkx* package. Spearman’s rank correlation coefficient was used to calculate correlations between parameters since their relationship was monotonical but not necessarily linear [41]. Also, Spearman’s rank correlation coefficient is less affected by the presence of outliers than Pearson’s correlation coefficient. To assess the statistical significance of the topological parameters, for each type of network in our study (MINs, DNs, MI clusters and MI3D clusters), a rewired model consisting of 1000 networks was built, where edges were randomly exchanged and the degree of each node was unchanged. All statistical calculations were performed using the R statistical programming language [40].

### Prediction of Functional Sites

We extracted the information on relevant single-residue positions of the sequence from UniProt, as it appears in the *feature* (FT) section [42]. Since the positions of UniProt entries are relative to unique protein sequences (and not to Pfam domains), we associated those functional features identified in >10% of the sequences used to build the MSA to a Pfam. The UniProt sequence features are defined as: MUTAGEN (site which has been experimentally altered, usually affecting protein activity); MOD_RES (site undergoing post-translational modification); METAL (binding site for a metal ion); BINDING (binding site for any chemical group, such as co-enzymes, prosthetic groups, etc.); ACT_SITE (site involved in the catalytic activity of the enzyme); SITE (any interesting single amino acid site on the sequence that is not defined by another feature key). We obtained features for 168 Pfams, averaging 1.7 features per Pfam.

We defined the baseline probability (P_Pfam_) of picking one residue of interest (e.g. a catalytic residue) in a Pfam domain by chance as the number of catalytic residues divided by the total number of residues of the domain. The probability of picking one catalytic residue within any of the MI clusters (P_MI_) was defined as the number of catalytic residues divided by the total number of residues in the MI clusters of the Pfam domain. Thus, the odds ratio (odds ratio = P_MI_/P_Pfam_) shows the increase in the likelihood of finding a catalytic residue in a MI cluster as compared to random chance. Likewise, the probability of picking one residue of interest (e.g. a catalytic residue) within any of the MI3D clusters of a Pfam (P_3D_) was defined as the number of catalytic residues divided by the total number residues on the MI3D clusters of that Pfam. Thus, the odds ratio (odds ratio = P_3D_/P_Pfam_; P_Pfam_ defined as above) shows the increase in the likelihood of finding a catalytic residue in a MI3D cluster as compared to random chance. The statistical association between a feature and a cluster of residues was quantified by means of a Fisher’s exact test, using 1000 sequences where the features had been randomly placed as a random model.

In order to associate features to topological parameters of the residues (namely, degree and clustering coefficient), we binned the values into three groups: SMALL, MEDIUM and LARGE, using the 33^th^ and 66^th^ percentile of the distribution of the degree and clustering coefficient values for each Pfam as thresholds. For MI clusters, we obtained the odd-ratio of the ranges by calculating the frequency of each feature over the all residues in the MI clusters as baseline (P_MI_) and the frequency of featured residues amongst those within a particular degree range (P_MI_^K^). The odds ratio was P_MI_^K^/P_MI_. A similar procedure was applied for the binned clustering coefficient (odds-ratio = P_MI_^c^/P_MI_). For MI3D clusters, the odds ratio was calculated as P_3D_^K^/P_3D_ and P_3D_^C^/P_3D_.

### Performance Analysis

We evaluated the performance of all different combinations of betweenness, relative degree and relative cluster size values as predictors of catalytic MI3D clusters in the 3DCN. For each 3DCN, we calculated relative degree of each cluster as the degree divided by the average degree of the 3DCN. Relative cluster size was calculated as the size of the cluster (number of residues) divided by the average cluster size of the 3DCN. We did so in order to make the parameters comparable across all Pfams. We used the F-measure as a measure of performance. It was defined as the harmonic mean of sensitivity and specificity: (2×sensitivity×specificity)/(sensitivity + specificity), where sensitivity is the ratio of correctly predicted catalytic MI3D clusters vs all predicted catalytic MI3D clusters and specificity is the ratio of correctly predicted non-catalytic MI3D clusters vs all predicted non-catalytic MI3D clusters.

## Supporting Information

Figure S1 **MIN and DN for the Pfam family PF00884.** (A) Representation of the MI network of the PF00884 family. Colours other than grey indicate the eight largest MI clusters. (B) Representation of the distance network calculated from the PDB structure 1AUK chain A, representative of the PF00884 family. Residues are coloured as in A.
(PNG)

Figure S2 **Distribution of modularity of MI clusters and MI3D clusters.** Fraction of Pfam families versus the modularity of the partition. Blue: partition of MI clusters (mean = 0.461). Orange: partition of MI3D clusters (mean = 0.359). Modularity values larger than 0 indicate a partition of the network resulting in denser clusters than random expectation.
(TIFF)

Figure S3 **Distribution of the number of MI clusters and MI3D clusters.** Thick line: distribution of the number of MI clusters (mean = 11.97). Thin line: distribution of the number of MI3D clusters (mean = 10.47).
(TIFF)

Figure S4 **Size distribution of MI and MI3D clusters.** Blue dots: size distribution of MI clusters (mean = 13.53; median = 6). Orange dots: size distribution of MI3D clusters (mean = 13.25; median = 6).
(TIFF)

Figure S5 **Distribution of the number of MI3D clusters.** Orange line: distribution of the number of catalytic MI3D clusters (mean = 3.40, median = 3). Blue line: distribution of the number of non-catalytic MI3D clusters (mean = 7.28, median = 7.28).
(TIFF)

Figure S6 **Ratio of the number of non-catalytic MI3D clusters vs catalytic MI3D clusters.** Ratio of the number of non-catalytic MI3D clusters vs catalytic MI3D clusters per Pfam (mean = 2.65, median = 2).
(TIFF)

Figure S7 **Size ratio of catalytic MI3D clusters vs non-catalytic MI3D clusters.** Size ratio of catalytic MI3D clusters vs non-catalytic MI3D clusters of the same Pfam (mean ratio = 4.12, median = 2.97).
(TIFF)

Figure S8 **Fraction of co-evolving residues between catalytic MI3D clusters.** The fraction of co-evolving residues between catalytic MI3D clusters is compared to random expectation by means of a *z*-score. The higher frequency of negative *z*-scores means that, for most Pfams, co-evolution between catalytic MI3D clusters is smaller than expected by chance.
(TIFF)

Figure S9 **Origin of catalytic and non-catalytic MI3D clusters.** Blue line: fraction of all MI clusters in a Pfam which produce non-catalytic MI3D clusters when mapped onto space (only Pfams with at least one non-catalytic MI3D cluster considered). Orange line: fraction of all MI clusters in a Pfam which produce catalytic MI3D clusters (only Pfams with at least one catalytic MI3D cluster considered). Black line: fraction of all MI clusters in a Pfam which produce both catalytic and non-catalytic MI3D clusters (only Pfams with catalytic and non-catalytic MI3D clusters considered). For 76.8% of the Pfams with catalytic and non-catalytic MI3D clusters, no MI clusters produce both catalytic and non-catalytic MI3D clusters when mapped onto space, with significantly less than random expectation (97.6%), KS test *p*-value = 9.67*10^−11^ (consequently, for 23.2% of Pfams there is at least one MI cluster which produces both catalytic and non-catalytic MI3D clusters). 10% of MI clusters out of 17.6% of Pfams produce catalytic and non-catalytic MI3D clusters.
(TIFF)

Figure S10 **Network of MI3D clusters and mapping onto the protein structure of PF01979.** (A) 3DCN of the Pfam family PF01979 (pdb code: 1KRA). Catalytic MI3D clusters were coloured yellow, orange and cyan. Clusters with less than 10 residues were coloured grey. The size of a node is proportional to the number of residues in the MI3D cluster. (B) Ribbon representation of the MI3D clusters of the representative structure of PF1979. Catalytic residues represented as red balls and sticks. (C) Surface representation of the view in B. (D) Same as A rotated 180 degrees. (E) Surface representation of the view in D.
(PDF)

Figure S11 **Network of MI3D clusters and mapping onto the protein structure of PF00118.** A) 3DCN of the Pfam family PF00118 (pdb code: 1A6D). Catalytic MI3D clusters were coloured yellow, orange, green and blue. Clusters with less than 10 residues were coloured grey. The size of a node is proportional to the number of residues in the MI3D cluster. (B) Ribbon representation of the MI3D clusters of the representative structure of PF00118. Catalytic residues represented as red balls and sticks. (C) Surface representation of the view in B. (D) Same as A rotated 180 degrees. (E) Surface representation of the view in D.
(PDF)

Figure S12 **Ratio of betweenness centrality in the MI3D cluster network.** Ratio of the betweenness centrality of catalytic MI3D clusters vs non-catalytic MI3D clusters within the same Pfam (mean = 12.89; median = 6.5).
(TIFF)

Figure S13 **Flowchart of the clustering process.** (A) In the MIN, residues are connected if they share a MI value >6 (black lines); in the DN, residues are connected if they are closer than 5 Å (red lines). (B) The MCL clustering algorithm identified *MI clusters* according to their density of connections. (C) MI clusters are mapped onto the 3D space of the protein, forming *MI3D clusters* (note that their connectivity pattern is no longer based on their MI values but on their physical distance). (D) MI3D clusters are connected if any of their residues are close in space, forming a MI3D cluster network.
(PDF)

Table S1 **OMIM mutations within MI3D clusters.** Mutations described by OMIM database in human proteins and their corresponding mutated position in the reference sequence. All mutations but one fall within a catalytic MI3D cluster.
(XLS)

Table S2 **Definition of topological parameters.** A local parameter characterizes a single node. A global parameter characterizes the whole network.
(PDF)

Text S1 **Small-world characteristics in MINs and DNs.** Calculation of clustering coefficient (*C*) and characteristic path length (*L*) for random networks and regular networks. Comparison of MINs and DNs to random and regular networks highlights their small-world structure.
(PDF)

Text S2 **Spatial clustering of Distance Networks.** Description of the clustering process of DNs to obtain spatial clusters. Evaluation of the performance of the prediction of functional residues using spatial clusters.
(PDF)
