# Supplementary figures and images for: Mapping the Mutual Information Network of Enzymatic Families in the Protein Structure to Unveil Functional Features
Source: PLoS One. 2012 Jul 25;7(7):e41430. doi: 10.1371/journal.pone.0041430 (PMC3405127; doi:10.1371/journal.pone.0041430)

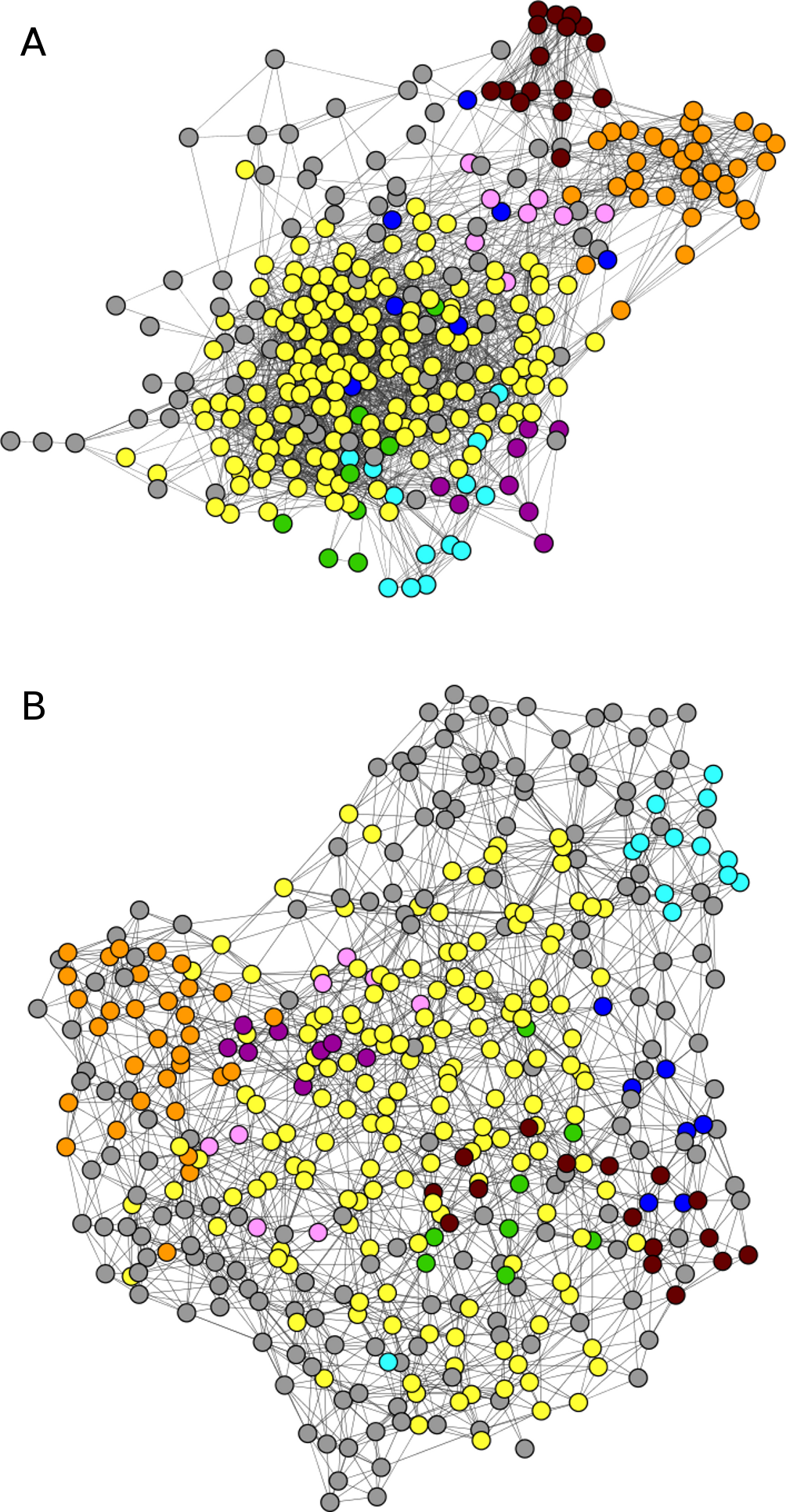

Supplement: Figure S1 — MIN and DN for the Pfam family PF00884. (A) Representation of the MI network of the PF00884 family. Colours other than grey indicate the eight largest MI clusters. (B) Representation of the distance network calculated from the PDB structure 1AUK chain A, representative of the PF00884 family. Residues are coloured as in A. (PNG) [file pone.0041430.s001.png]

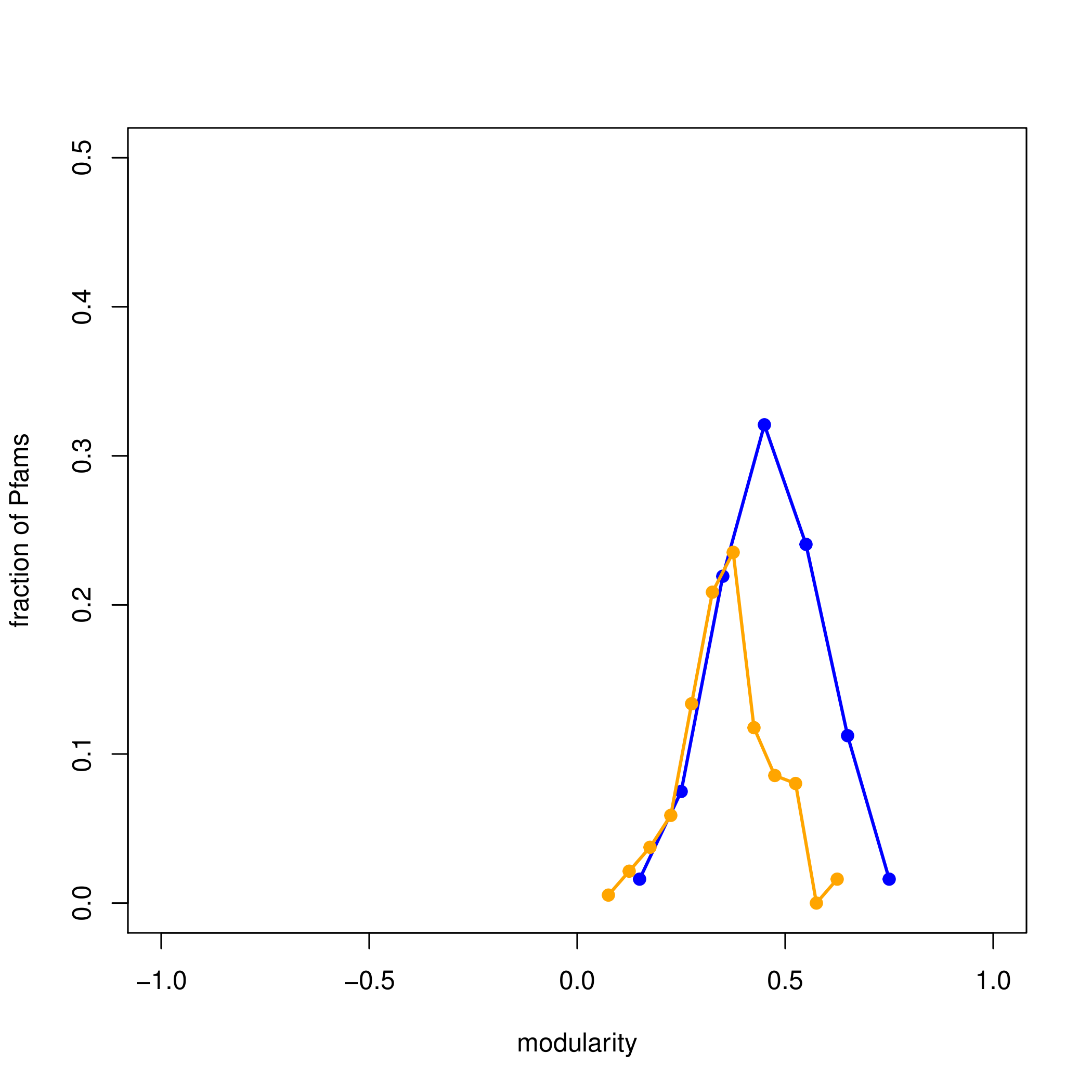

Supplement: Figure S2 — Distribution of modularity of MI clusters and MI3D clusters. Fraction of Pfam families versus the modularity of the partition. Blue: partition of MI clusters (mean = 0.461). Orange: partition of MI3D clusters (mean = 0.359). Modularity values larger than 0 indicate a partition of the network resulting in denser clusters than random expectation. (TIFF) [file pone.0041430.s002.tiff]

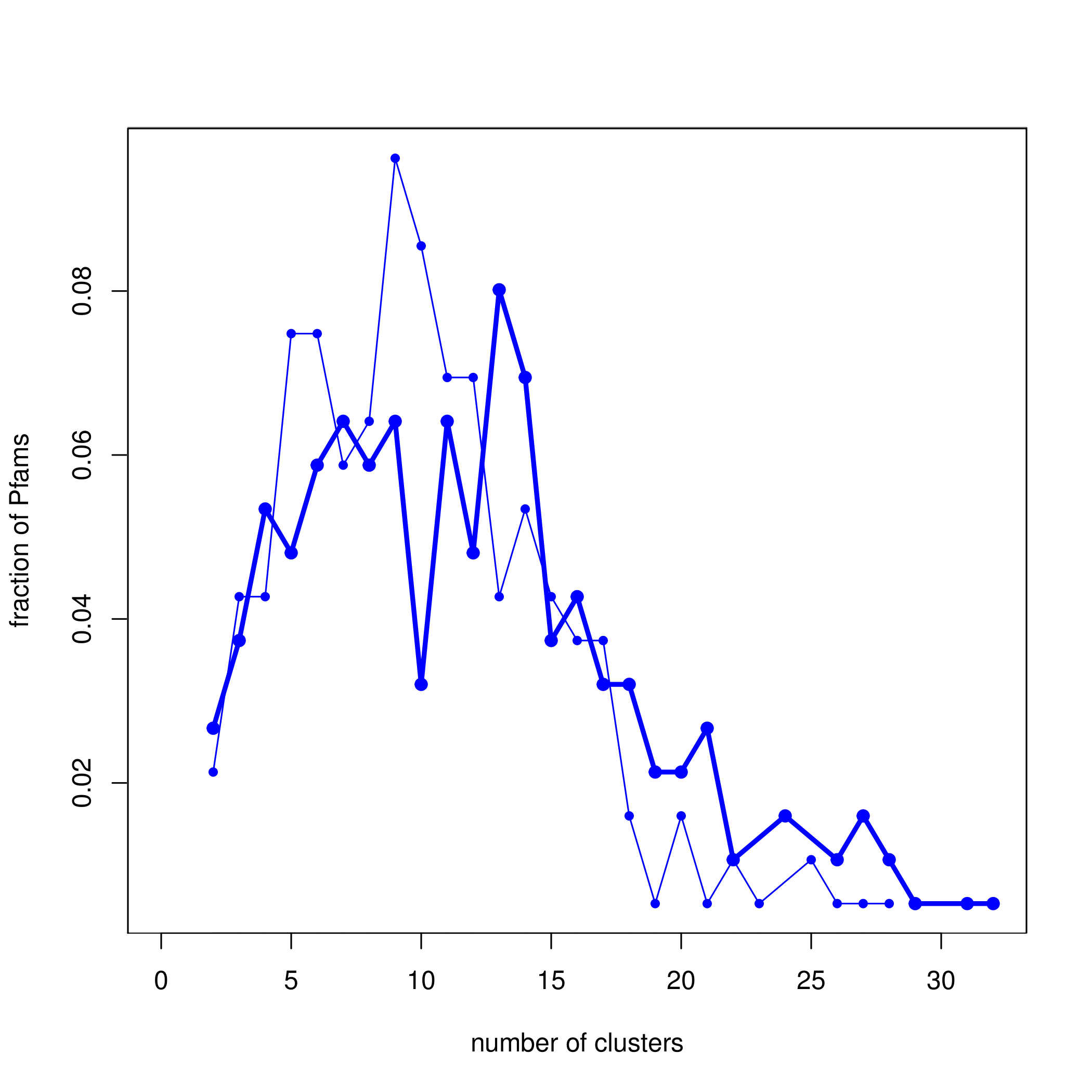

Supplement: Figure S3 — Distribution of the number of MI clusters and MI3D clusters. Thick line: distribution of the number of MI clusters (mean = 11.97). Thin line: distribution of the number of MI3D clusters (mean = 10.47). (TIFF) [file pone.0041430.s003.tiff]

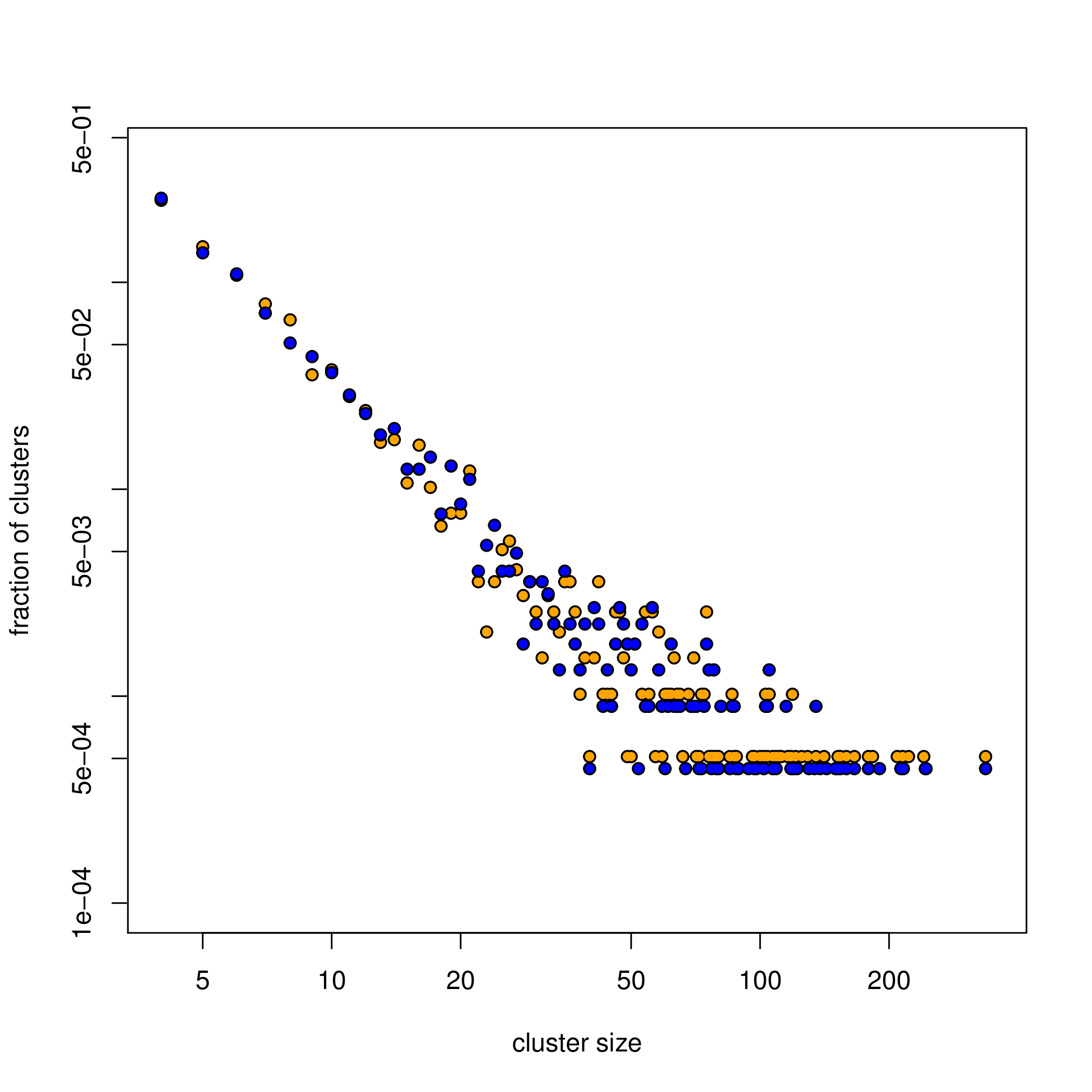

Supplement: Figure S4 — Size distribution of MI and MI3D clusters. Blue dots: size distribution of MI clusters (mean = 13.53; median = 6). Orange dots: size distribution of MI3D clusters (mean = 13.25; median = 6). (TIFF) [file pone.0041430.s004.tiff]

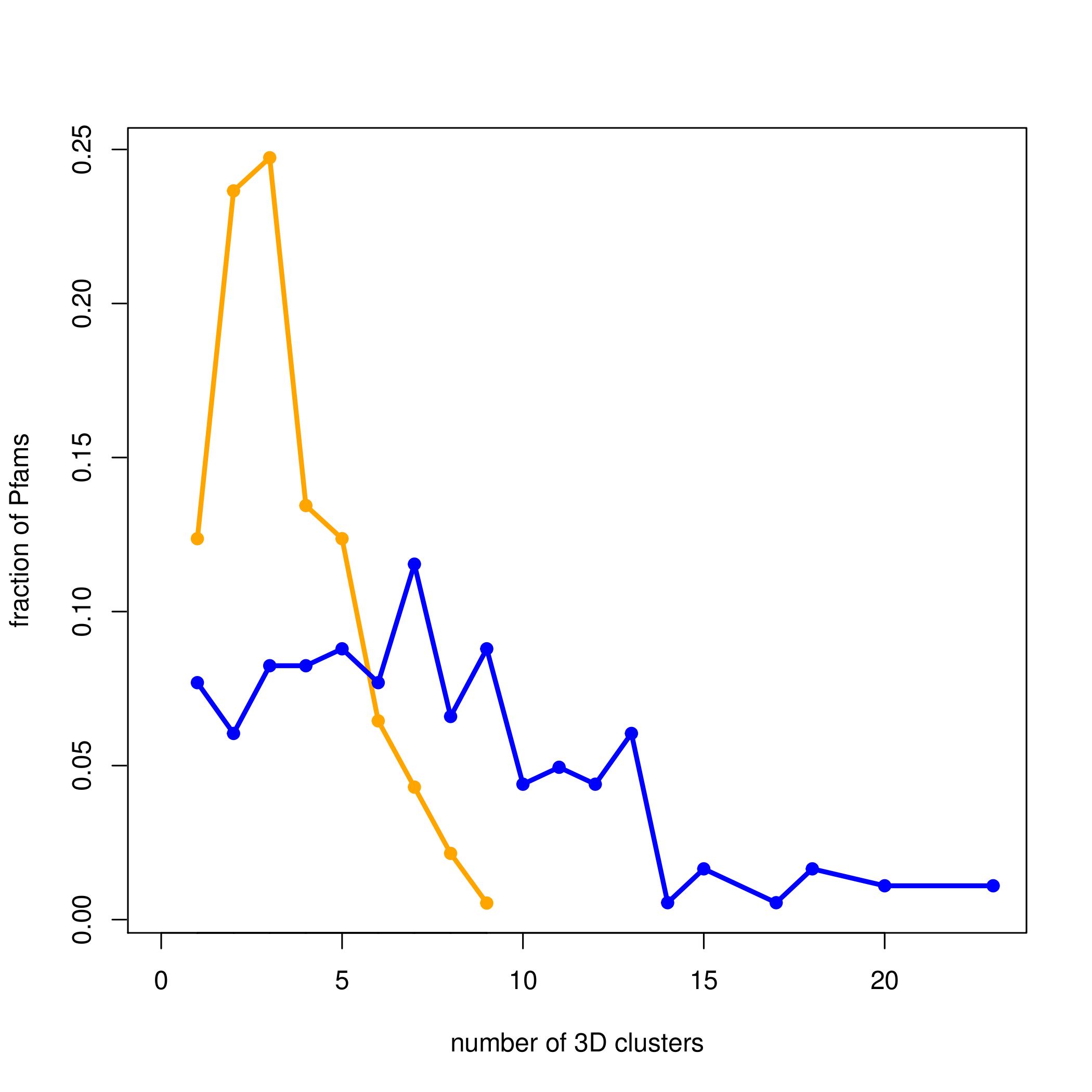

Supplement: Figure S5 — Distribution of the number of MI3D clusters. Orange line: distribution of the number of catalytic MI3D clusters (mean = 3.40, median = 3). Blue line: distribution of the number of non-catalytic MI3D clusters (mean = 7.28, median = 7.28). (TIFF) [file pone.0041430.s005.tiff]

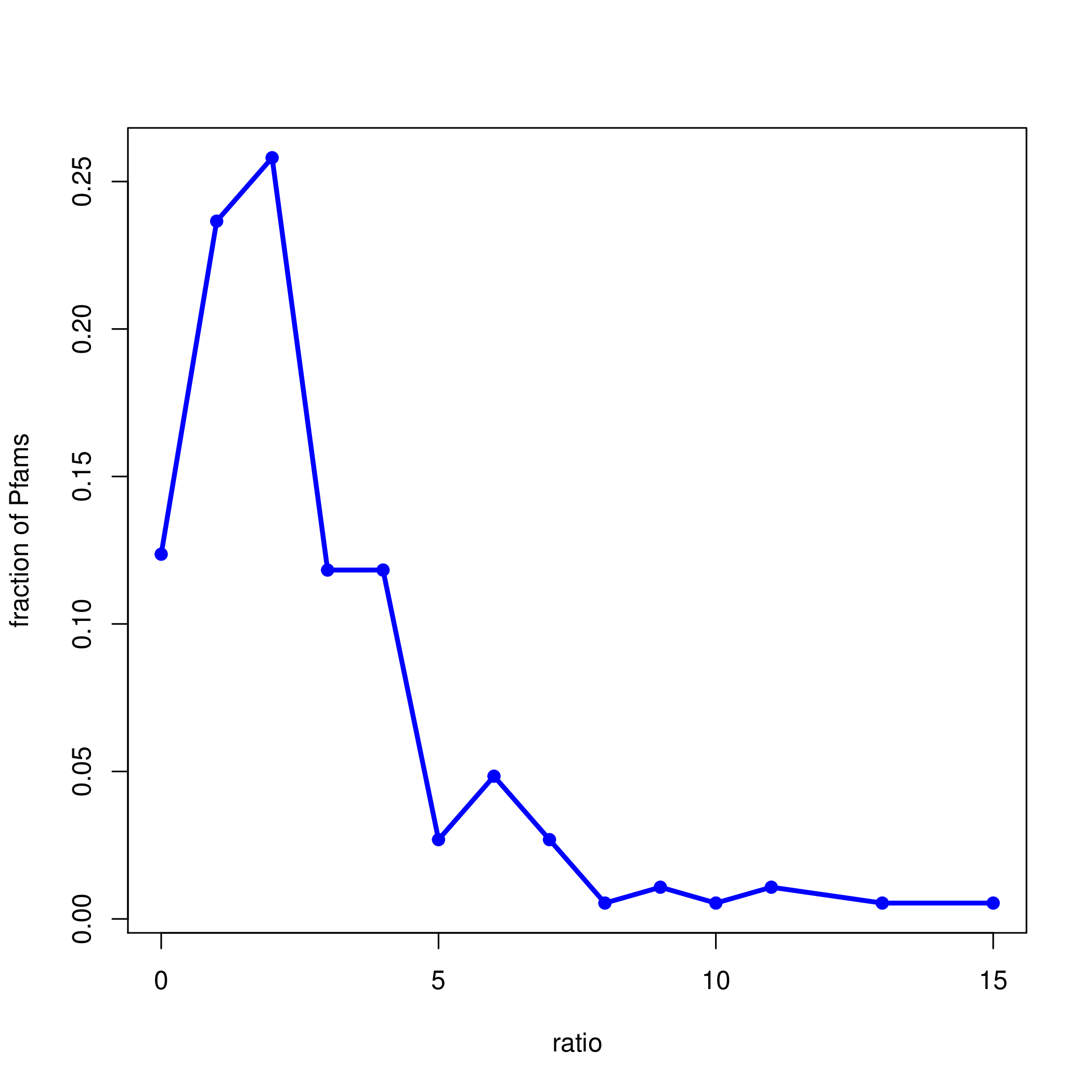

Supplement: Figure S6 — Ratio of the number of non-catalytic MI3D clusters vs catalytic MI3D clusters. Ratio of the number of non-catalytic MI3D clusters vs catalytic MI3D clusters per Pfam (mean = 2.65, median = 2). (TIFF) [file pone.0041430.s006.tiff]

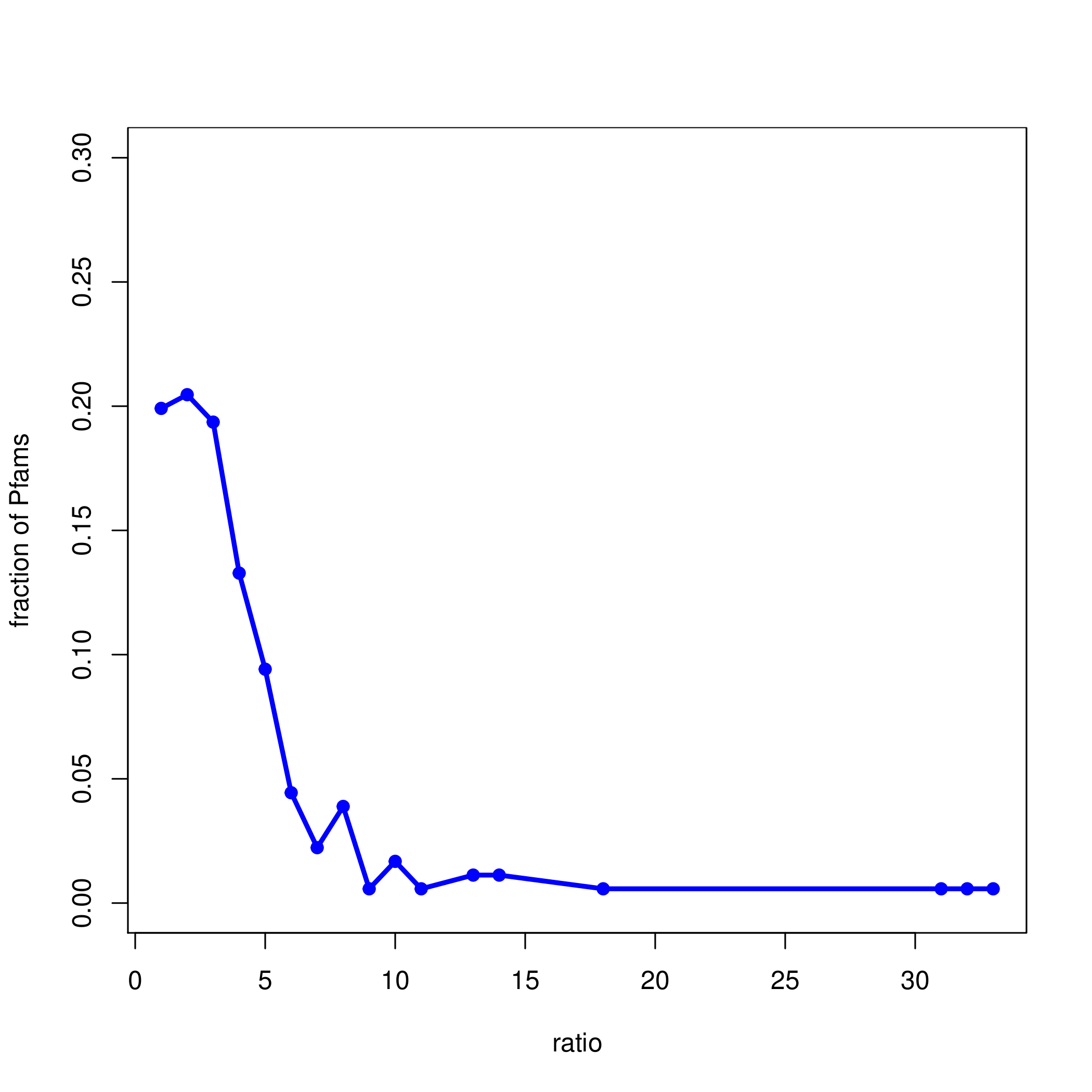

Supplement: Figure S7 — Size ratio of catalytic MI3D clusters vs non-catalytic MI3D clusters. Size ratio of catalytic MI3D clusters vs non-catalytic MI3D clusters of the same Pfam (mean ratio = 4.12, median = 2.97). (TIFF) [file pone.0041430.s007.tiff]

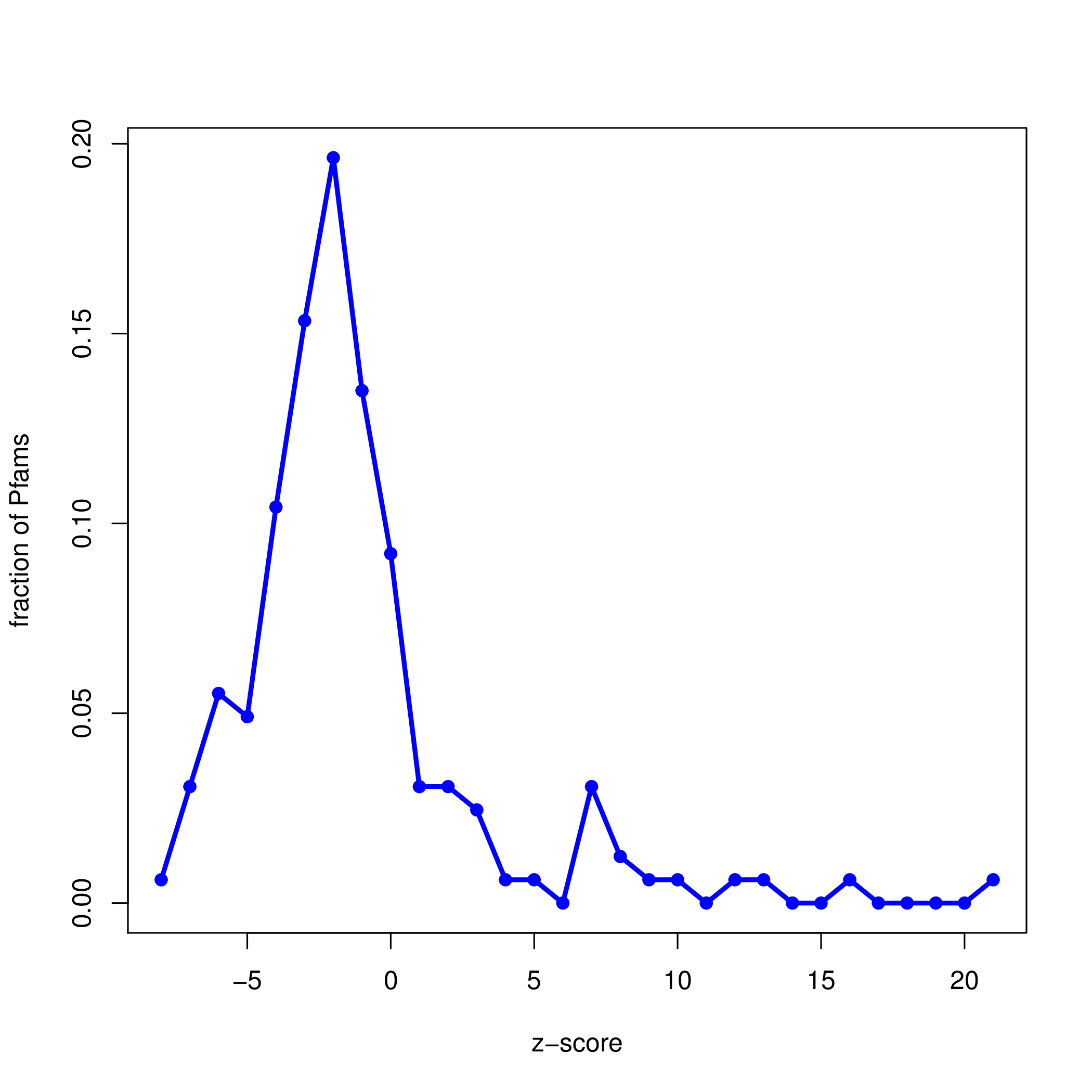

Supplement: Figure S8 — Fraction of co-evolving residues between catalytic MI3D clusters. The fraction of co-evolving residues between catalytic MI3D clusters is compared to random expectation by means of a z-score. The higher frequency of negative z-scores means that, for most Pfams, co-evolution between catalytic MI3D clusters is smaller than expected by chance. (TIFF) [file pone.0041430.s008.tiff]

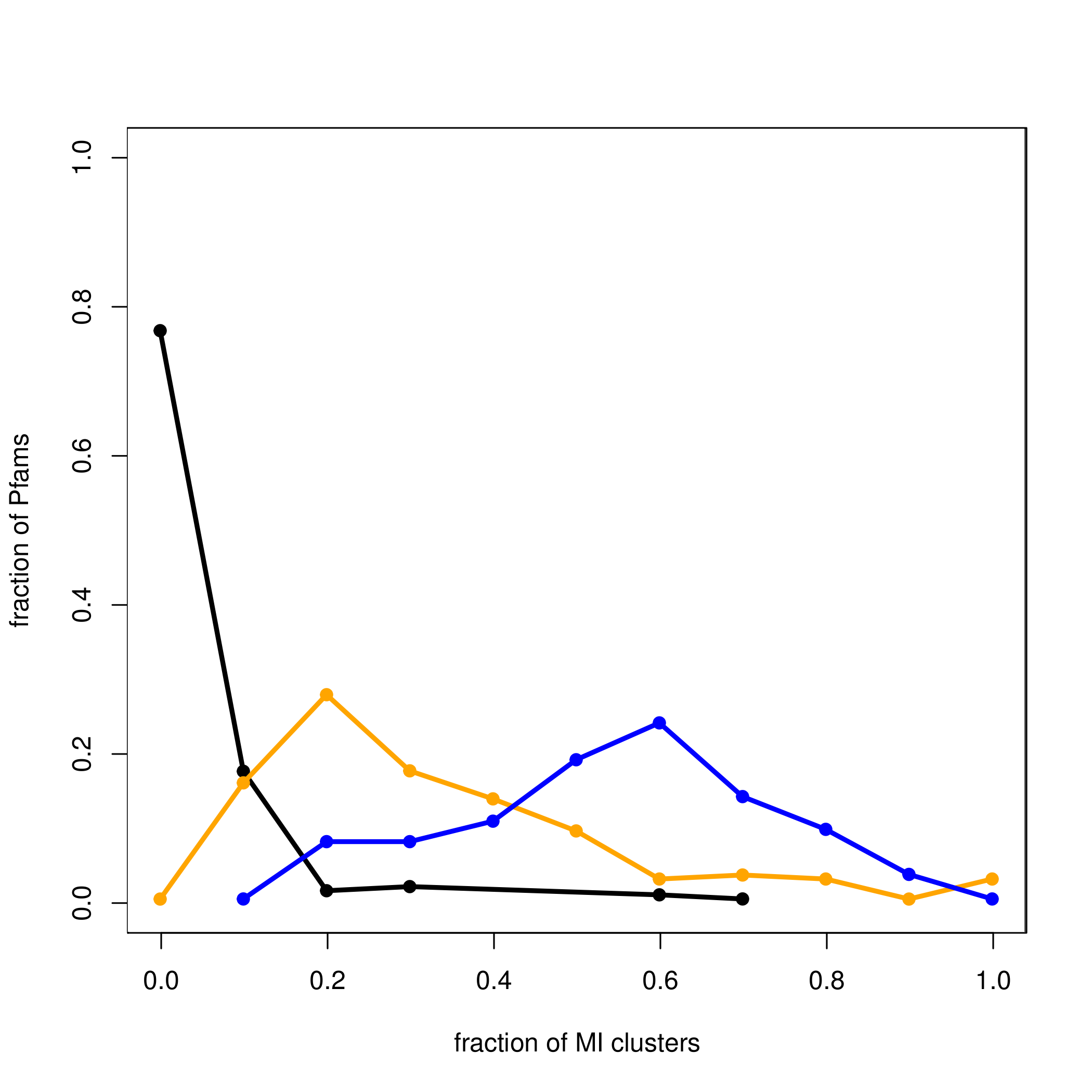

Supplement: Figure S9 — Origin of catalytic and non-catalytic MI3D clusters. Blue line: fraction of all MI clusters in a Pfam which produce non-catalytic MI3D clusters when mapped onto space (only Pfams with at least one non-catalytic MI3D cluster considered). Orange line: fraction of all MI clusters in a Pfam which produce catalytic MI3D clusters (only Pfams with at least one catalytic MI3D cluster considered). Black line: fraction of all MI clusters in a Pfam which produce both catalytic and non-catalytic MI3D clusters (only Pfams with catalytic and non-catalytic MI3D clusters considered). For 76.8% of the Pfams with catalytic and non-catalytic MI3D clusters, no MI clusters produce both catalytic and non-catalytic MI3D clusters when mapped onto space, with significantly less than random expectation (97.6%), KS test p-value = 9.67*10−11 (consequently, for 23.2% of Pfams there is at least one MI cluster which produces both catalytic and non-catalytic MI3D clusters). 10% of MI clusters out of 17.6% of Pfams produce catalytic and non-catalytic MI3D clusters. (TIFF) [file pone.0041430.s009.tiff]

A

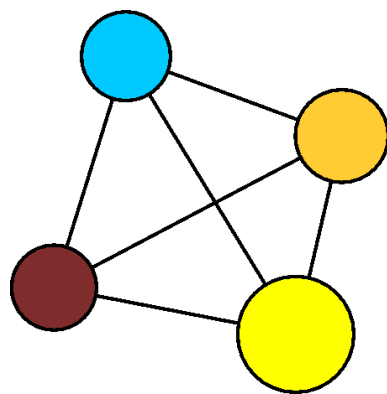

B

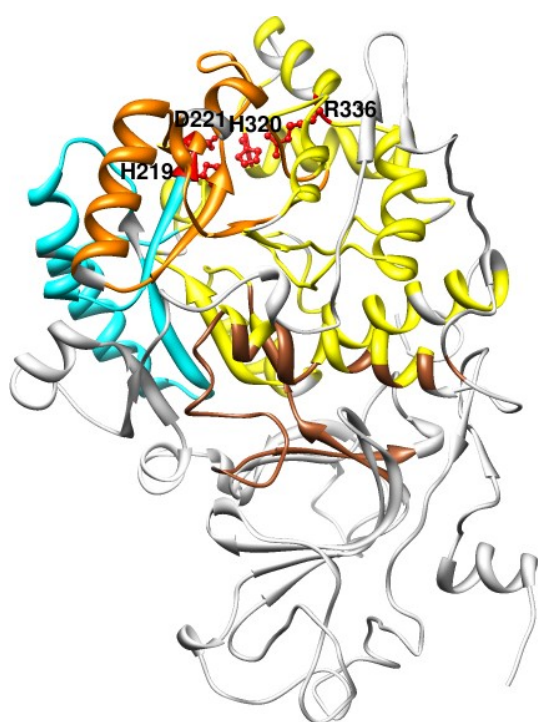

C

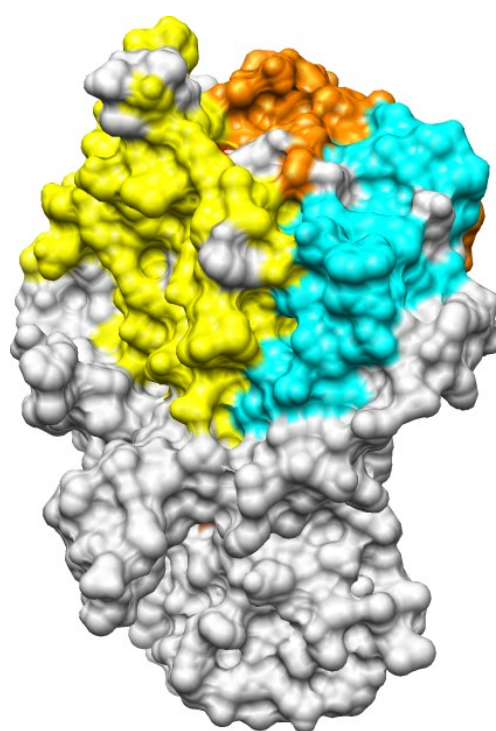

D

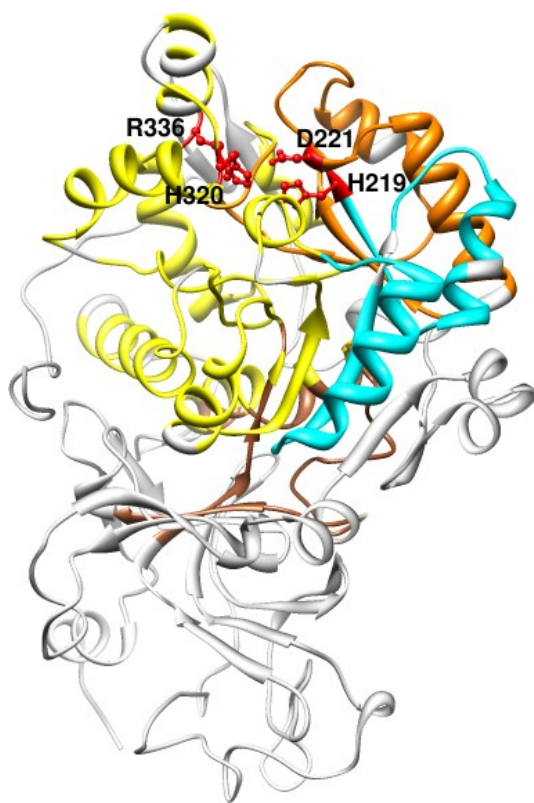

E

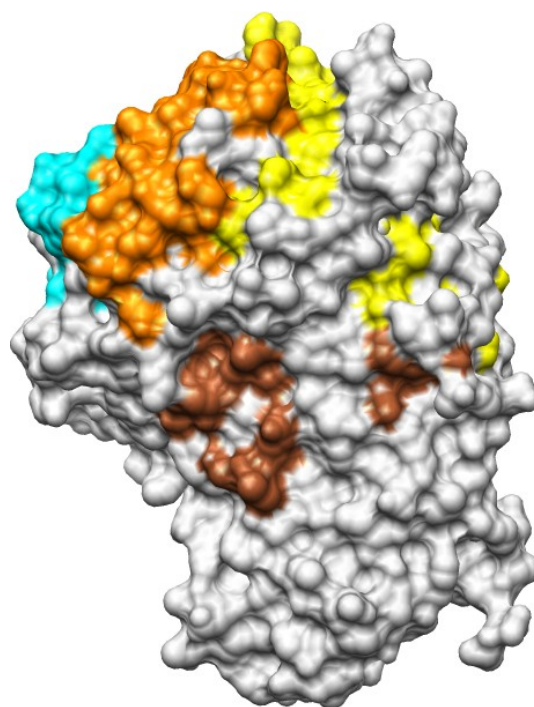

Supplement: Figure S10 — Network of MI3D clusters and mapping onto the protein structure of PF01979. (A) 3DCN of the Pfam family PF01979 (pdb code: 1KRA). Catalytic MI3D clusters were coloured yellow, orange and cyan. Clusters with less than 10 residues were coloured grey. The size of a node is proportional to the number of residues in the MI3D cluster. (B) Ribbon representation of the MI3D clusters of the representative structure of PF1979. Catalytic residues represented as red balls and sticks. (C) Surface representation of the view in B. (D) Same as A rotated 180 degrees. (E) Surface representation of the view in D. (PDF) [file pone.0041430.s010.pdf]

A

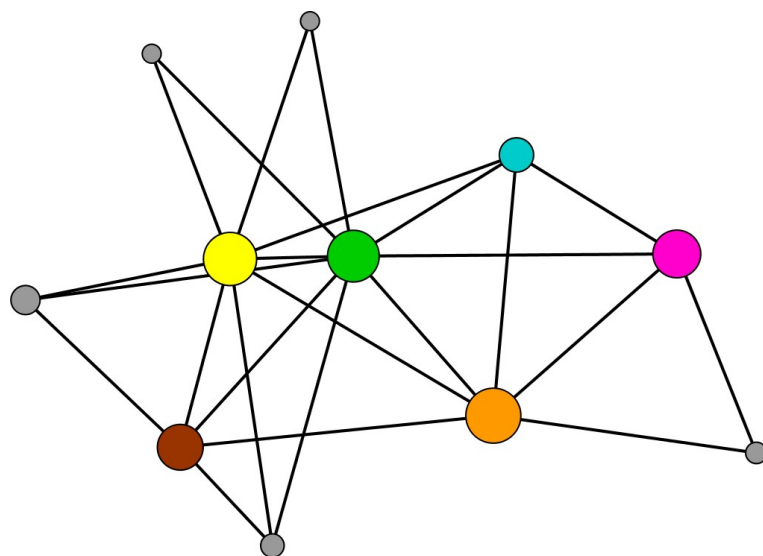

B

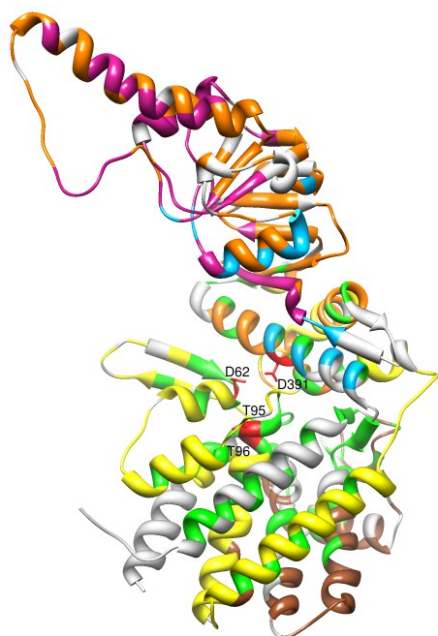

C

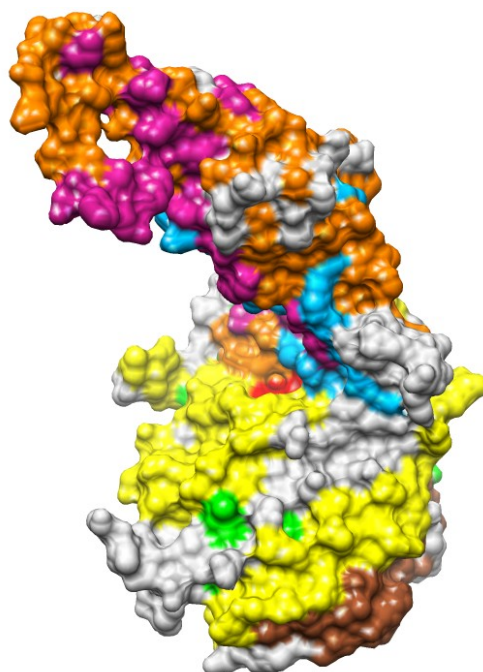

D

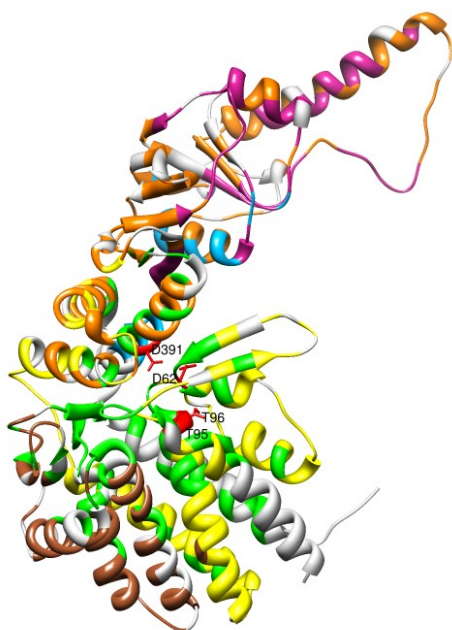

E

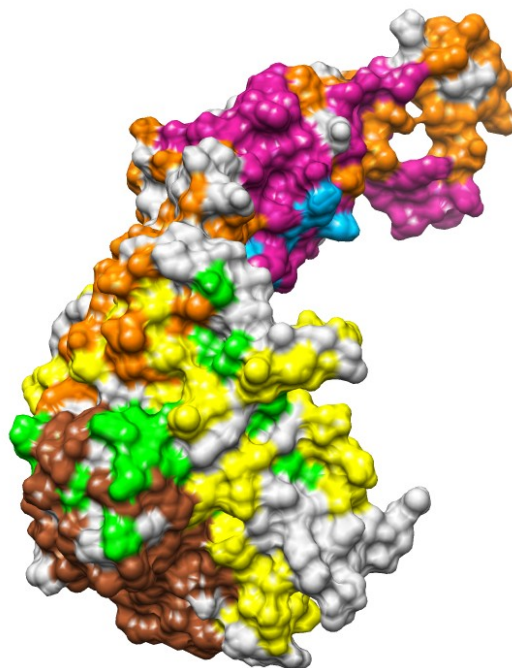

Supplement: Figure S11 — Network of MI3D clusters and mapping onto the protein structure of PF00118. A) 3DCN of the Pfam family PF00118 (pdb code: 1A6D). Catalytic MI3D clusters were coloured yellow, orange, green and blue. Clusters with less than 10 residues were coloured grey. The size of a node is proportional to the number of residues in the MI3D cluster. (B) Ribbon representation of the MI3D clusters of the representative structure of PF00118. Catalytic residues represented as red balls and sticks. (C) Surface representation of the view in B. (D) Same as A rotated 180 degrees. (E) Surface representation of the view in D. (PDF) [file pone.0041430.s011.pdf]

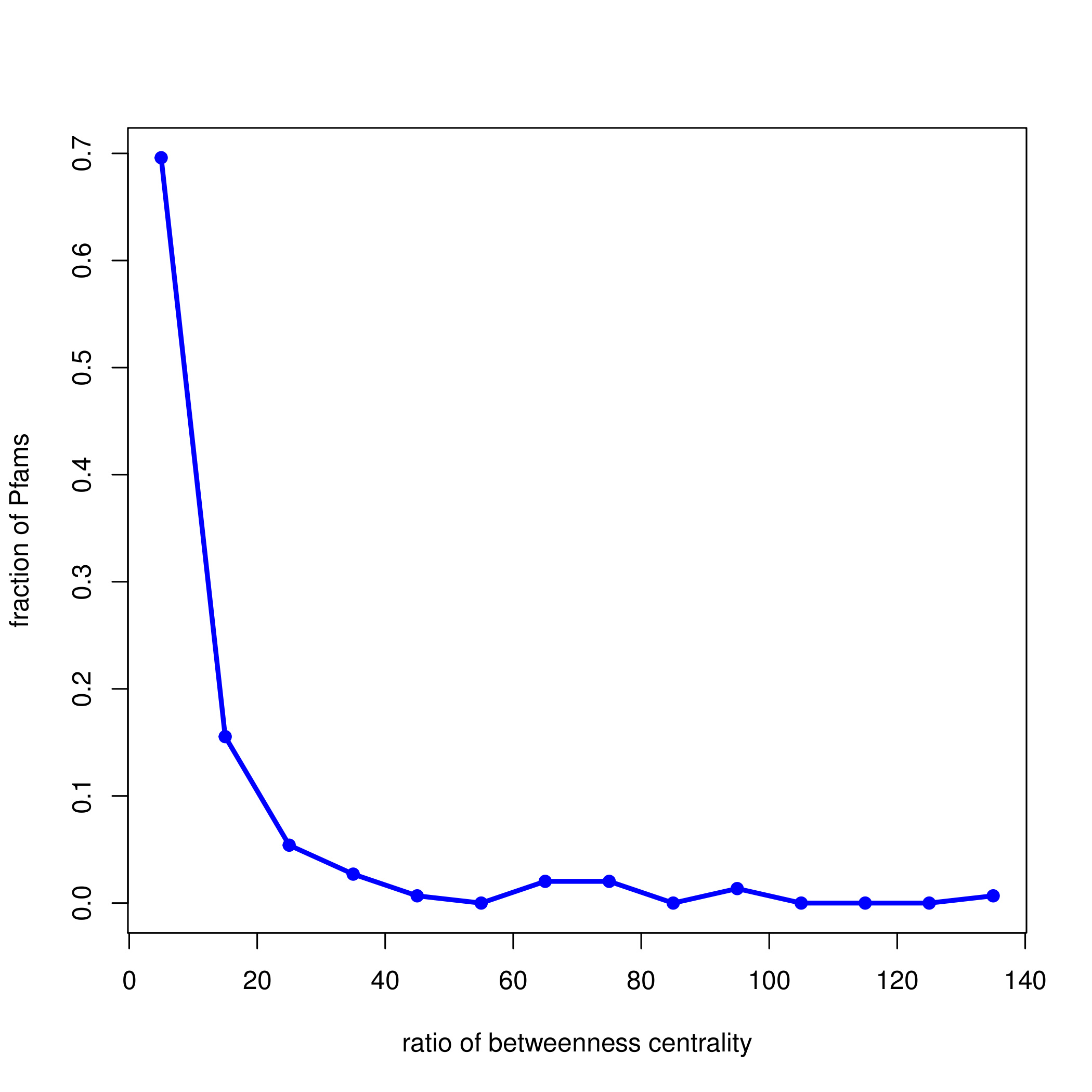

Supplement: Figure S12 — Ratio of betweenness centrality in the MI3D cluster network. Ratio of the betweenness centrality of catalytic MI3D clusters vs non-catalytic MI3D clusters within the same Pfam (mean = 12.89; median = 6.5). (TIFF) [file pone.0041430.s012.tiff]

A

Mutual information data

3D structure data

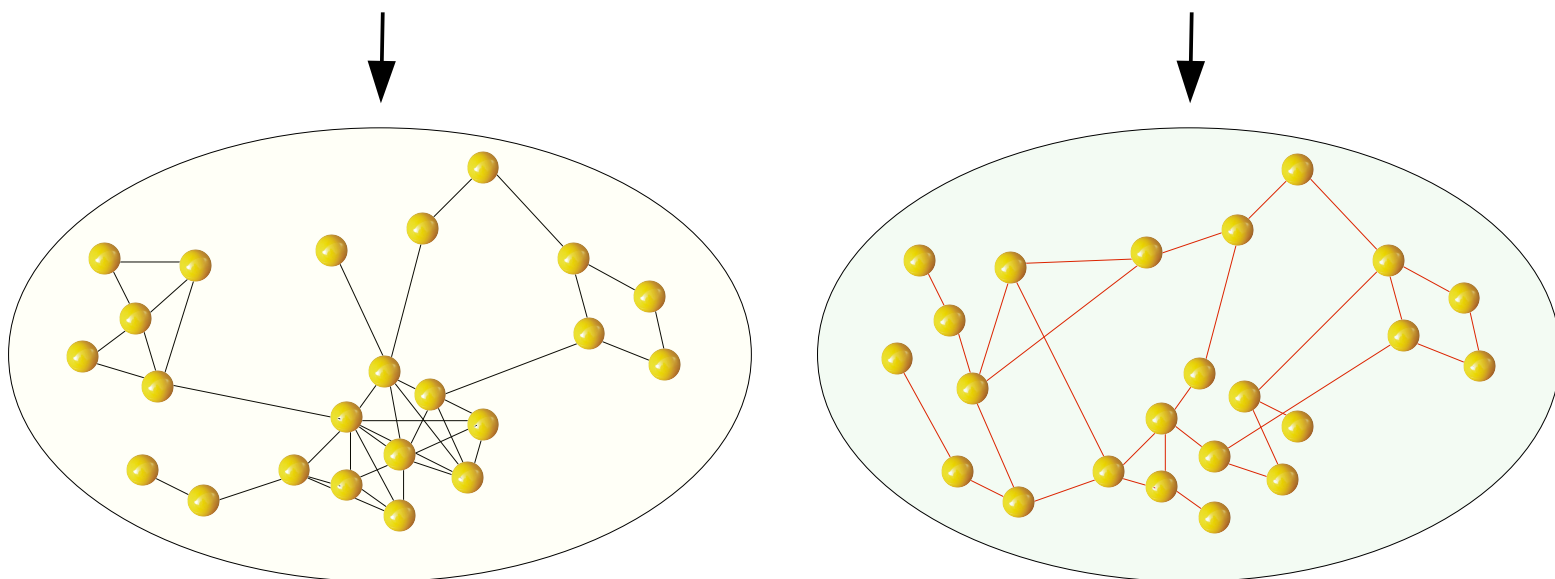

B

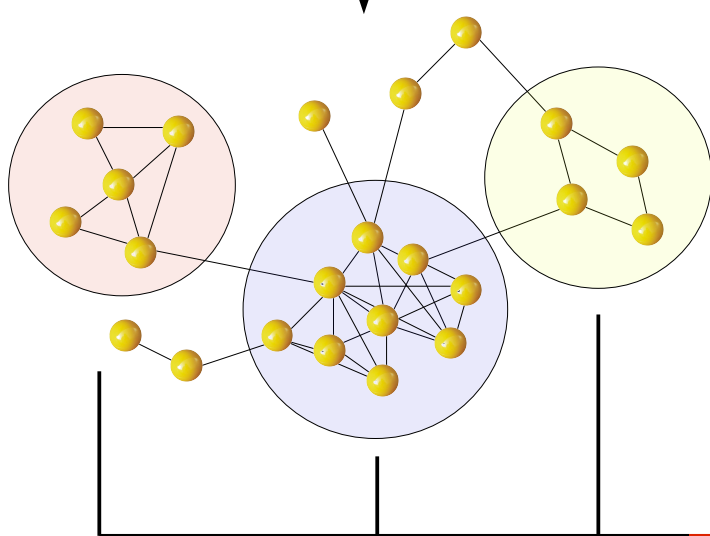

C

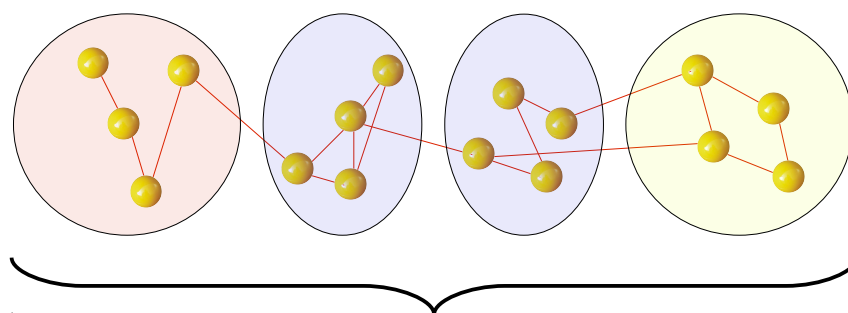

D

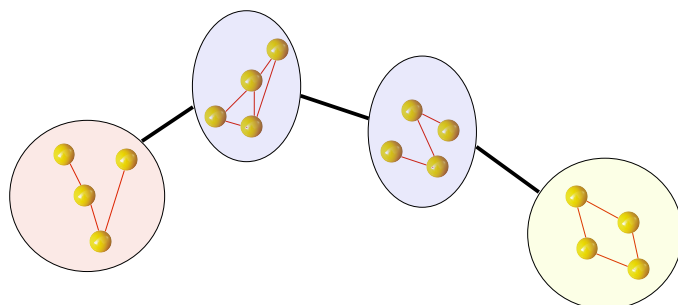

Supplement: Figure S13 — Flowchart of the clustering process. (A) In the MIN, residues are connected if they share a MI value >6 (black lines); in the DN, residues are connected if they are closer than 5 Å (red lines). (B) The MCL clustering algorithm identified MI clusters according to their density of connections. (C) MI clusters are mapped onto the 3D space of the protein, forming MI3D clusters (note that their connectivity pattern is no longer based on their MI values but on their physical distance). (D) MI3D clusters are connected if any of their residues are close in space, forming a MI3D cluster network. (PDF) [file pone.0041430.s013.pdf]
