# Supplementary material for: Mapping the Mutual Information Network of Enzymatic Families in the Protein Structure to Unveil Functional Features
Source: PLoS One. 2012 Jul 25;7(7):e41430. doi: 10.1371/journal.pone.0041430 (PMC3405127; doi:10.1371/journal.pone.0041430)
Supplement: Table S2 — Definition of topological parameters. A local parameter characterizes a single node. A global parameter characterizes the whole network. (PDF) [file pone.0041430.s015.pdf]

| Parameter | Definition |
| --- | --- |
| Degree | Number of connections of a node (i.e. number of neighbors of a node). Local parameter. |
| Clustering coefficient | Ratio of the number of connections in the neighborhood of a node and the total number of connections if the neighborhood was fully connected. Local parameter. |
| Density | Ratio of the number of connections in the network and the total number of connections if the network was fully connected. Global parameter. |
| Characteristic path length | Number of steps of the shortest path between any two nodes averaged for all pairs of nodes in the network. Global parameter. |
| Modularity | The modularity of a division of network is the fraction of the edges included in the resulting groups minus the expected such fraction if edges were randomly distributed. Global parameter. |
| Betweenness centrality | Ratio of the number of shortest paths between any two nodes in the network that pass through a given node. Local parameter. |
