# Supplementary material for: Mapping the Mutual Information Network of Enzymatic Families in the Protein Structure to Unveil Functional Features
Source: PLoS One. 2012 Jul 25;7(7):e41430. doi: 10.1371/journal.pone.0041430 (PMC3405127; doi:10.1371/journal.pone.0041430)
Supplement: Text S1 — Small-world characteristics in MINs and DNs. Calculation of clustering coefficient (C) and characteristic path length (L) for random networks and regular networks. Comparison of MINs and DNs to random and regular networks highlights their small-world structure. (PDF) [file pone.0041430.s016.pdf]

SUPPLEMENTARY TEXT S1

Small-world characteristics in MINs and DNs

METHODS

We calculated average clustering coefficient (*C*) and characteristic path length (*L*) for MINs and DNs as explained in the main manuscript. For comparison, we computed the corresponding values in random and regular networks as follows [1]: C_{random} = K/N, L_{random} = ln N/ln K , $C_{regular}$ = 3(K-2)/[4(K-1)] and $L_{regular}$ = N(N+K-2)/[2K(N-1)], where K is the number of nodes and N is the number of edges in the network.

RESULTS

In their seminal work, Watts and Strogatz describe the small-world phenomenon as characterized by two parameters: $L >= L_{random}$ and $C >> C_{random}$ [2]. Figures 1 and 2 in this text show that MINs and DNs are both small world networks, largely showing this behaviour.

As for comparison with regular networks, our results for DNs agree with the observations of Vendruscolo *et al.* for protein structures [1]. Results for MINs are similar to those observed by Chakrabarti and Panchenko [3]. The only difference is that, in our case, $C < C_{regular}$ in most MINs (95.6%), while in theirs $C > C_{regular}$ on average. Two possible explanations for this discrepancy are: (1) Chakrabarti and Panchenko used a lower number of aligned sequences in order to calculate MI values (which can be source of bias [4]), and (2) a different method for mutual information threshold calculation was used, which in our case produced sparser networks.

REFERENCES

1. Vendruscolo M, Dokholyan NV, Paci E, Karplus M (2002). Small-world view of the amino acids that play a key role in protein folding. Phys Rev E Stat Nonlin Soft Matter Phys. 65: 061910.
2. Watts DJ, Strogatz SH (1998). Collective dynamics of 'small-world' networks. Nature 393: 409–10.
3. Chakrabarti S, Panchenko AR (2010). Structural and functional roles of coevolved sites in proteins. PloS one 5: e8591.
4. Dunn SD, Wahl LM, Gloor GB (2007). Mutual information without the influence of phylogeny or entropy dramatically improves residue contact prediction. Bioinformatics 24: 333–340.

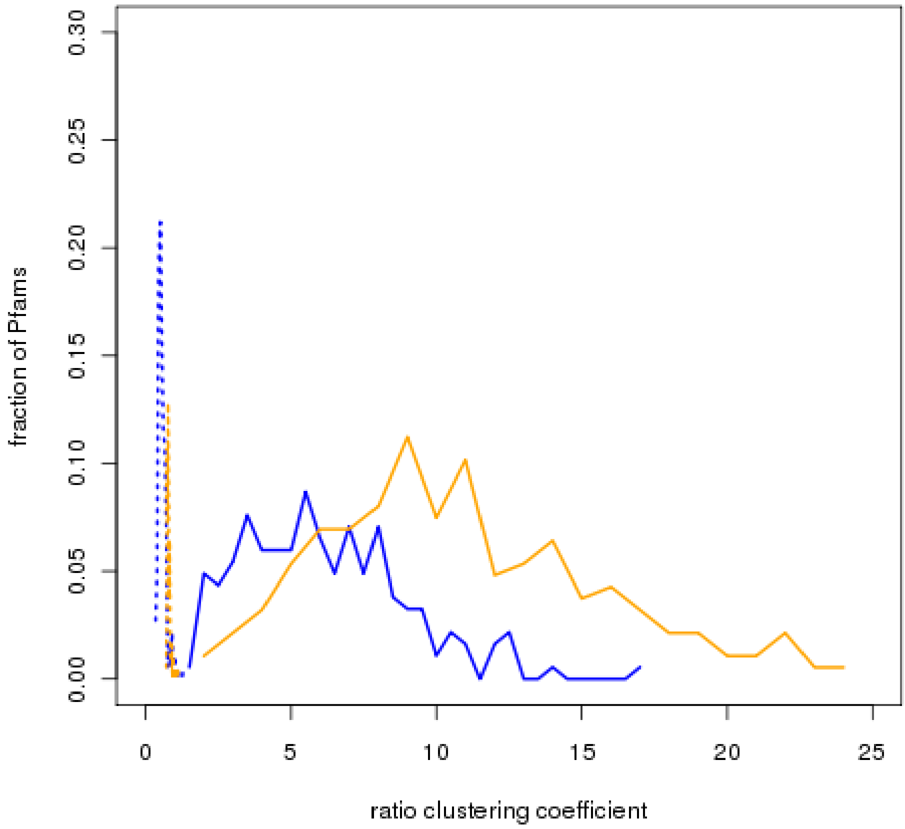

Figure 1. Distribution of ratios of C/C_{random} for MINs (blue solid line), $C/C_{regular}$ for MINs (blue dotted line), C/C_{random} for DNs (orange solid line), $C/C_{regular}$ for DNs (orange dotted line).

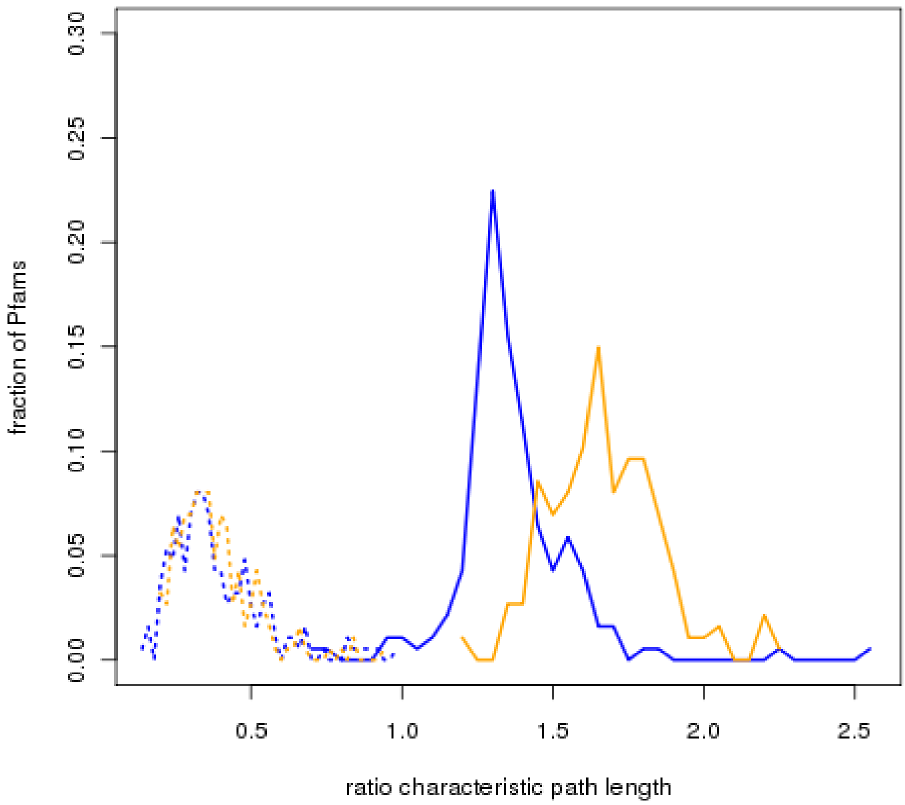

Figure 2. Distribution of ratios of L/L_{random} for MINs (blue solid line), $L/L_{regular}$ for MINs (blue dotted line), L/L_{random} for DNs (orange solid line), $L/L_{regular}$ for DNs (orange dotted line).
