# Supplementary material for: Mapping the Mutual Information Network of Enzymatic Families in the Protein Structure to Unveil Functional Features
Source: PLoS One. 2012 Jul 25;7(7):e41430. doi: 10.1371/journal.pone.0041430 (PMC3405127; doi:10.1371/journal.pone.0041430)
Supplement: Text S2 — Spatial clustering of Distance Networks. Description of the clustering process of DNs to obtain spatial clusters. Evaluation of the performance of the prediction of functional residues using spatial clusters. (PDF) [file pone.0041430.s017.pdf]

SUPPLEMENTARY TEXT S2

Spatial clustering of Distance Networks

METHODS

We used the MCL clustering algorithm (see main manuscript) to cluster the DNs. The process resulted in each DN divided in 17 clusters on average, which contained groups of residues close in space (but which do not necessarily co-evolve). We called these clusters *spatial clusters.*

Next, for each clustered DN we built a network where two clusters were linked if they were in physical contact (<5 Å) by at least one residue (the clustering process is density-based, so it is possible for two separated clusters to still have a certain amount of contacting residues). The process is equivalent to that we used to build 3DCNs. We evaluated the performance of the prediction of catalytic clusters using this network of spatial clusters.

RESULTS

We compared the value of these spatial clusters for the prediction of functional residues. Results are shown as odds ratios in table 1.

Compared to table 1 in the main manuscript, spatial clusters have worse prediction value. Only the feature MUTAGEN is statistically over-represented in spatial clusters, whilst MI clusters and 3D clusters were enriched in ACT_SITE, MUTAGEN, METAL and BINDING features. Functional enrichment in spatial clusters associated to degree and clustering coefficient is only significant for the ACT_SITE feature, whist for MI clusters was significant for METAL, ACT_SITE and BINDING features.

As for the network of spatial clusters, we used the size, betweenness centrality and degree of the network of spatial clusters to predict which ones were catalytic (i.e. contained at least one catalytic residues). With optimal thresholds for the three predictors (relative size=0.2, betweenness=0.1, relative degree=1.0), we achieved a 66.4 F-measure with spatial clusters, which is slightly worse than the result that we obtained using the 3DCN (F-measure = 73.2).

	**Spatial clusters**	**Small *k***	**Med. *k***	**Large *k***	**Signif.**	**Small *C***	**Med. *C***	**Large *C***	**Signif.**
**MOD_RES**	1.12	1.13	1.42	1.82		1.39	2.67	3.27	
**MUTAGEN**	4.61 *	1.76	1.26	3.06		2.11	2.04	3.68	
**METAL**	1.01	1.67	2.1	2.07		2.47	1.24	2.41	
**ACT_SITE**	1.09	1.59	2.78	2.44	*	1.24	2.61	3.45	*
**BINDING**	0.87	1.99	1.59	1.82		1.75	1.66	2.65	
**SITE**	1.51	4.44	1.51	2.37		1.85	1.28	4.1	

Table 1. The *spatial clusters* column show the odds ratio of finding functional residues in spatial clusters. Small *k*, medium *k* and large *k* show odds ratio for residues with small, medium and large degree, respectively, on spatial clusters. Small *C*, medium *C* and large *C* show odds ratio for residues with small, medium and large clustering coefficient, respectively, on spatial clusters.
